# Dissolving Microneedles Developed in Association with Nanosystems: A Scoping Review on the Quality Parameters of These Emerging Systems for Drug or Protein Transdermal Delivery

**DOI:** 10.3390/pharmaceutics13101601

**Published:** 2021-10-02

**Authors:** Patrícia Weimer, Rochele Cassanta Rossi, Letícia Scherer Koester

**Affiliations:** 1 Programa de Pós-Graduação em Ciências Farmacêuticas, Faculdade de Farmácia, Universidade Federal do Rio Grande do Sul (UFRGS), Porto Alegre 90610-000, Brazil; patricia.weimer@ufrgs.br; 2 Programa de Pós-Graduação em Nutrição e Alimentos, Universidade do Vale do Rio dos Sinos (UNISINOS), São Leopoldo 93022-000, Brazil; rochelecr@unisinos.br

**Keywords:** microarray patch, dissolving microneedles, hydrogel-forming microneedles, nanostructured systems, nanoparticles, transdermal delivery, transcutaneous immunization, skin permeation, skin deposition, quality control

## Abstract

The largest organ of the body provides the main challenge for the transdermal delivery of lipophilic or high molecular weight drugs. To cross the main barrier of the skin, the stratum corneum, many techniques have been developed and improved. In the last 20 years, the association of microneedles with nanostructured systems has gained prominence for its versatility and for enabling targeted drug delivery. Currently, the combination of these mechanisms is pointed to as an emerging technology; however, some gaps need to be answered to transcend the development of these devices from the laboratory scale to the pharmaceutical market. It is known that the lack of regulatory guidelines for quality control is a hindrance to market conquest. In this context, this study undertakes a scoping review of original papers concerning methods applied to evaluate both the quality and drug/protein delivery of dissolving and hydrogel-forming microneedles developed in association with nanostructured systems.

## 1. Introduction

In the last decades, there has been growing interest in the delivery of substances, drugs, and proteins by transdermal route owing to specific advantages of this route, including the absence of first-pass effect, reduction in the number of doses achieved by controlled release, and good acceptability by patients. For transdermal administration, compounds are usually delivered in semi-solid formulations or transdermal patches that allow them to penetrate and permeate through the layers of the skin to the blood capillaries (in the dermis) and, consequently, systemic circulation [1,2].

However, the transdermal route confers some limitations on the administration of classical pharmaceutical forms, restricting the administration of compounds according to lipophilicity and molecular size (<500 Da). To overcome these limitations, physical and chemical stimuli, such as permeation promoters, nanostructured systems, and microscopic applicators, have been investigated to aid in the permeation of compounds, especially in the transport to the stratum corneum (SC). The SC is an outer skin barrier composed of proteins and lipids that physiologically hinders the entry of external agents into the skin and prevents transepidermal water loss [1,2,3]. Recently, some studies have pointed out that besides the SC, the thickness of the epidermis is a determining factor in the process of permeation of substances [4]. In this context, transdermal devices that release the compounds directly into the epidermis and even close to the dermis considerably decrease these limitations [5,6,7,8].

First introduced for transdermal administration in 1998 [9], the microarray patches or microneedles (MNs) have made a name for themselves owing to their remarkable versatility. The reduced height of the MN and adjustments in geometric conformation allow painless application to the epidermis or close to the dermis without reaching the pain receptors and allow the administration of substances of different lipophilicity [10,11]. In addition, through technological modifications that have enabled the advancement from metallic MN to polymeric MN design, MNs have been named as one of the top ten emerging technologies in 2020 and have become a market promise for the possibility of drug, protein, and hormone delivery [12,13,14]. The global transdermal drug delivery system (TDDS) market is projected to be worth USD 8.4 billion by 2027, at a compound annual growth rate (CAGR) of 4.3% [15], and projections for the global market for MNs in drug delivery systems comprise a CAGR of 6.6% between 2020 and 2030, representing an estimated market value of USD 1.2 billion by 2030 [16].

The development of polymeric MNs, in addition to expanding the possibilities of application of these devices, have also allowed the proposal of one-step administration, in which the loaded compound is dispersed in the MN [17]. To enhance the effects of the loaded substances and protect against possible degradation, nanostructured systems have been studied in association with MNs [18,19]. Certain characteristics of nanostructured systems can be applied to circumvent the disadvantages of MNs, such as low drug loading capacity. The increased surface area guaranteed by nanostructures makes it possible to obtain similar biological effects with the application of lower doses of the drugs compared to the bulk form. In addition, nanostructures can be designed to model the release rate or biodistribution profile of drugs or proteins according to the pathophysiological microenvironment, for example, insulin release at high blood glucose levels. In technological terms, nanostructures may contribute to modulating the mechanical characteristics of microneedles, increasing mechanical strength and contributing to cutaneous insertion, for example [20,21,22,23].

Recent reviews have emphasized the benefits of the association of MNs with nanostructured systems, especially for the release of hydrophobic substances [11,24,25,26]. Furthermore, these studies have pointed out that despite the promising market for MNs and their association with nanostructured systems, there are still gaps that hinder the transposition from bench to industrial scale. The main barriers associated with the clinical feasibility and production of microneedles include the evaluation of the characteristics after scaling up production, sterilization processes that do not damage the device, clinical evaluation of human safety and immunogenic potential, patient acceptability rate, evaluations of the pharmacokinetic and pharmacodynamic properties of the drugs administered by these systems, and the lack of regulatory guidelines on quality control [26,27,28,29]. Among them, the lack of regulatory guidelines on quality control impacts the scale-up process and the success rate of this market.

In this context, this review maps the quality control methods frequently applied to polymeric MNs (dissolution and hydrogel-formation) designed for transcutaneous delivery of substances, drugs, or proteins carried in nanostructured systems.

## 2. Methods

### 2.1. Protocol and Registration

The search protocol was drafted based on the protocol guidance of the Preferred Reporting Items for Systematic Review and Meta-Analysis Protocols (PRISMA-P) [30] and was registered in the Open Science Framework on 12 June 2021 (https://osf.io/j5mxu (accessed on 13 June 2021)).

### 2.2. Information Sources and Search Strategy

The studies were searched in June 2021 in three databases: Web of Science, EMBASE, and MEDLINE (PubMed), in which the search was restricted to English only, with no additional restriction on publication date or document type. The search strategy was composed of three-word queries combined with the Boolean operator “AND”. The first query presented terms related to microneedles, the second related to nanostructured systems, and the third was composed of terms associated with the transdermal release of substances and drugs. All terms were searched in titles, abstracts, and keywords, and minor modifications were made according to database specifications. The detailed search strategies are presented in the Supplementary Materials (Table S1). In addition, the search results were exported in excel or CSV format and grouped in a single spreadsheet to remove duplicates.

### 2.3. Selection of Sources of Evidence

Prior to defining the databases and search strategy, a pilot search was conducted to evaluate the suitability of the queries and search terms as well as the specificity and sensitivity of the search strategy. In addition, two articles were used as indicators of specificity and selectivity as they fulfilled all the inclusion criteria and described the complete quality control analyses that were to be retrieved after searching the databases. Complementarily, these articles were also defined as search quality markers for evaluating the application of a natural compound and a synthetic drug already established in the market, respectively, comprising two search objectives of this review. The ACS Publications database was also considered for the search; however, the pilot search identified the retrieval of the same articles in the other databases, and, therefore, it was excluded after this step.

### 2.4. Eligibility Criteria

To be included in the review, studies were assessed for exclusion and inclusion criteria in two stages: the eligibility of titles and abstracts and the eligibility of full texts. After the exclusion of duplicate studies, the titles and abstracts were analyzed, and articles fitting the following criteria were excluded: (a) review articles, book chapters, or conference abstracts; (b) articles without reference to the use of microneedles; (c) articles without reference to the application of nanostructured systems; (d) administration by another route and not transdermal, or (e) development of a device for diagnosis or sensing. The remaining articles were evaluated for full-text content, and those meeting the following criteria were excluded: (a) application of MNs only for skin pretreatment (microporation technique), (b) development of solid, hollow, and coated MNs, (c) no description of the nanostructured system, (d) substance not associated with nanostructures, (e) no target delivery of either substances, drugs, or proteins, (f) no description of quality control assays of MNs, (g) not in accordance with the scope of the review (e.g., cell delivery), and (h) no access to the full text.

In the case of no access to the full articles or need for additional information, the authors of the original articles were contacted. In the absence of a response, the respective articles were excluded from the review.

### 2.5. Data Items and Data Extraction

The data from the studies were extracted into spreadsheets, including descriptive variables (year of publication, authors, country of origin, category of delivery, category of study, objective of device application, type of MN and nanosystem, polymeric composition of the device, and MN geometry), and data from the quality control tests of the microarray patch were divided into two categories: *in vitro* and *in vivo* assays. Specific data were extracted from the *in vitro* assays: content analysis, mechanical properties, insertion assay, dissolution and release profile, and permeation and distribution profiles. Additionally, data from stability studies were extracted. Regarding *in vivo* assays, data from skin insertion, skin release/permeation, and skin dissolution were extracted.

Secondary data such as nanostructured system preparation method, device production method, and *in vivo* efficacy trials were extracted to support the discussion section.

### 2.6. Synthesis of the Results

The results were grouped according to the main categories of quality control assays, including *in vitro* and *in vivo* assays. Studies that showed similar patterns were summarized in graphs, and descriptive variables were summarized in tables. The final wording of the results followed the criteria established by the PRISMA-ScR guide for scoping reviews [31].

## 3. Results

### 3.1. Selection and General Characteristics of Sources of Evidence

**Figure 1:**
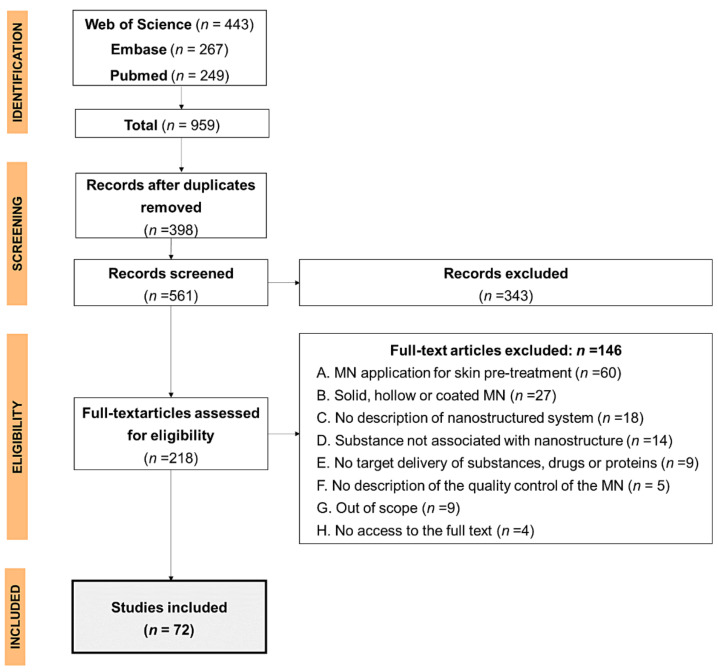
Flowchart of the included studies. MN: microneedles.

The application of the search strategy (Table S1) retrieved 959 studies published between 2001 and 2021, from which duplicate studies were excluded (*n* = 398), leaving 561 studies for analysis of eligibility of titles and abstracts. Of these, 343 studies were excluded because they were review articles, book chapters, or conference abstracts (*n* = 211), did not employ microneedles (*n* = 53) or nanostructured systems (*n* = 38), or targeted mucosal administration (*n* = 21) or sensing or diagnosis (*n* = 20). The flowchart of the study selection process is shown in Figure 1.

**Figure 2:**
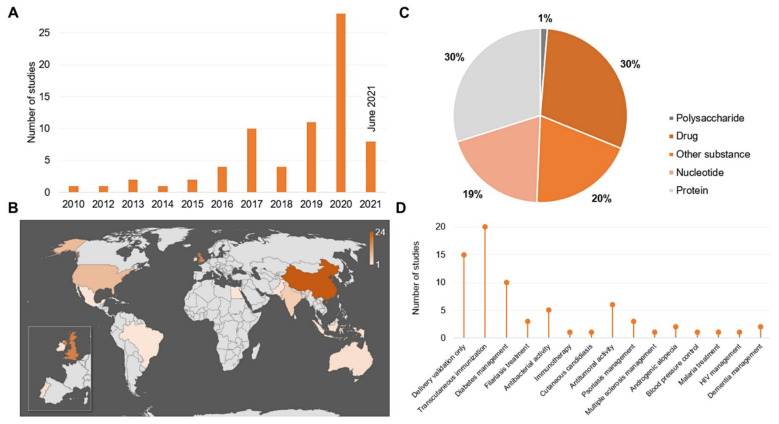
Characteristics of the included studies. (**A**) Number of articles by year of publication. (**B**) Number of articles by country of origin; in detail, United Kingdom and Portugal. (**C**) Delivery category. (**D**) Objective of the proof of concept.

As shown in Figure 1, of the 218 full-text articles analyzed, 146 were excluded for not meeting the inclusion criteria, leaving 72 articles for the review. Although the search retrieved articles from 2001 onwards, the included articles showed annual distribution from 2010 onwards (Figure 2A). Prior to this period, the studies employed the MNs as a pre-treatment for the microporation of the skin, which was subsequently exposed to either a patch or semisolid formulation containing the drug. When grouping the studies by country of origin (Figure 2B), it was revealed that the largest number of publications were from China (*n* = 24), followed by the UK (*n* = 17) and USA (*n* = 8), with minimal participation of publications from South America and Africa (*n* = 3).

Regarding the delivery category (Figure 2C), 30% of the studies developed the MNs with nanostructured systems for protein delivery, 30% for drug delivery, followed by 20% other substances and 19% nucleotide; only 1% described a device for polysaccharide delivery. With respect to protein and nucleotide devices, 43.5% were designed for insulin and 73.3% for DNA delivery, respectively. Additionally, the highest percentage of studies were conducted to validate a proof of concept for transcutaneous immunization (*n* = 20) or to validate the delivery mechanism of drugs and substances (*n* = 15), followed by diabetes management (*n* = 10), as shown in Figure 2D. Looking into the studies that evaluated antitumoral activity, the most used preclinical model comprised melanoma cell lines, accounting for 66.8% of this category [32,33,34,35,36]. Likewise, 60.0% of the studies that aimed at antibacterial activity applied the device in a biofilm model [37,38,39]. Overall, the results showed in Figure 2D suggest greater investment in diseases that affect the immune system, prioritizing transcutaneous immunization and the management of psoriasis [40,41,42], superficial tumors [32,33,34,35,36,43], and diseases that affect the lymphatic system owing to its proximity to the systemic circulation, such as filariasis [22,44,45]. Above all, diabetes mellitus, which requires constant insulin administrations, was prioritized [20,46,47].

### 3.2. Characteristics of Devices

**Figure 3:**
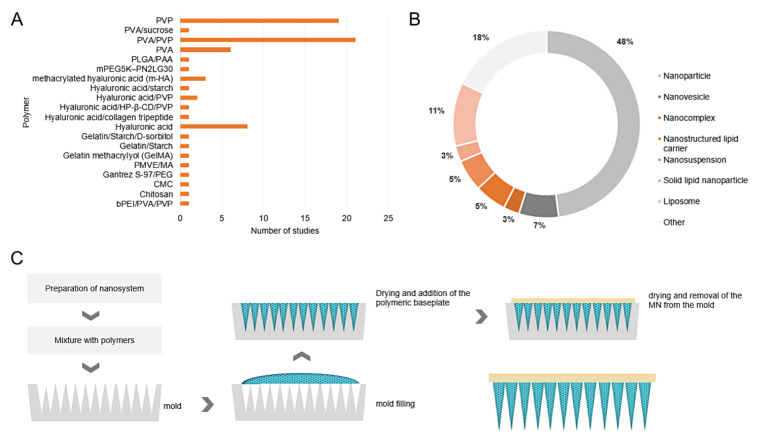
General characteristics of the developed microneedles. (**A**) Polymers employed in the devices. (**B**) Type of nanostructured system associated in the microarray patch. (**C**) Graphic representation of the method of obtaining microneedles by micromolding. PVP: polyvinylpyrrolidone; PVA: poly(vinyl alcohol); PVA/PVP/PVA: poly(vinyl alcohol)/polyvinylpyrrolidone; PLGA/PAA: poly(lactide-*co*-glycolide)/poly(acrylic acid), mPEG5K-PN2LG30: α-methoxy-poly(ethylene glycol)-l-glutamate, HP-β-CD: hydroxypropyl-β-cyclodextrin; PMVE/MA: copolymer of methylvinyl ether and maleic anhydride; PEG: poly (ethylene glycol); CMC: sodium carboxymethylcellulose.

Out of the 72 included studies, 71 articles described the preparation of dissolving MNs and 1 article the preparation of hydrogel-forming MNs [48]. Although this single study addressed a coated MN, it was included because it characterized a polymeric MN with a PLGA polymeric film containing the nanoparticles and not a coated solid-metallic MN, an exclusion criterion applied in the selection step of the studies. As presented in Figure 3A, the most frequently applied polymers in the preparation of the MN were polyvinylpyrrolidone (PVP), poly (vinyl alcohol)/polyvinylpyrrolidone (PVA/PVP), poly (vinyl alcohol) (PVA), and hyaluronic acid, representing approximately 74% of the total 20 polymers or polymer blends employed. Furthermore, the results revealed that most studies employed nanoparticles in association with MNs (51%), followed by liposomes (11%), and nanovesicles (7%) (Figure 3B). The other nanosystems with less representation, such as polymeric micelles, cubosomes, nanocrystals, and nanoclusters, were grouped in the category of other systems (18%). Among nanoparticles, the largest number of studies employed polymeric nanoparticles (51.4%), followed by mesoporous nanoparticles of different materials (13.5%) and amphipathic peptide (RALA) (13.5%), solid lipid (5.4%), metallic (5.4%), inorganic (5.4%), and gelatin nanoparticles (5.4%).

To obtain the devices, only one study employed the drawing lithography technique [18]; the other studies employed the micromolding method. This method consists of transferring the polymeric solution containing the nanostructures to a mold, usually composed of polydimethylsiloxane (PDMS), which is subsequently subjected to centrifugation or a vacuum chambering for the removal of possible bubbles and final drying [49,50,51,52]. Figure 3C demonstrates the graphical representation of this technique. Exceptionally, for obtaining hydrogel-forming MNs, the micromolding technique was combined with an electrohydrodynamic atomization process for coating with the nanoparticles. With reference to the shape and dimensions of the devices, most studies employed molds to obtain pyramidal and conical MNs, with minor exceptions for other shapes such as tanto-blade MNs, the design of which was inspired by traditional Japanese Samurai swords formed by two bevels [53], and only three studies did not inform the MN shape. The dimensions of the needles ranged from 323 to 1500 nm in height [50,54] and from 170 to 400 nm in base diameter [42,55], with the most frequent dimensions varying from 600 and 300 nm [19,56,57], respectively. Approximately 75% of the studies did not describe the needle tip diameter, which ranged from 5 to 40 nm [56,58]. Only four studies did not describe any MN dimension but confirmed the morphology of the MN by scanning electron microscopy (SEM) [23,59,60] or fluorescence microscopy [23,61]. Finally, regarding needle array, this variable ranged from 5 × 5 [45,62] to 33 × 33 array [42,63].

### 3.3. Methods Employed in Quality Control

The main methods applied for the characterization of devices composed of nanostructured systems associated with polymeric MNs are discussed in the sections below. Importantly, 26.4% of the studies were employed only in vitro, and 73.6% employed a combination of in vitro and in vivo methods. Since the aim of this review is to map the quality control methods in the analysis of this type of device, the in vivo trials are presented and discussed in line with the objective, as the models employed for disease evaluation are specific and indicate the effectiveness of the proposed treatment. Additionally, the characterization tests of the nanostructured systems before insertion into the polymeric matrix of MNs are presented in Figure S1 (Supplementary Materials).

**Figure 4:**
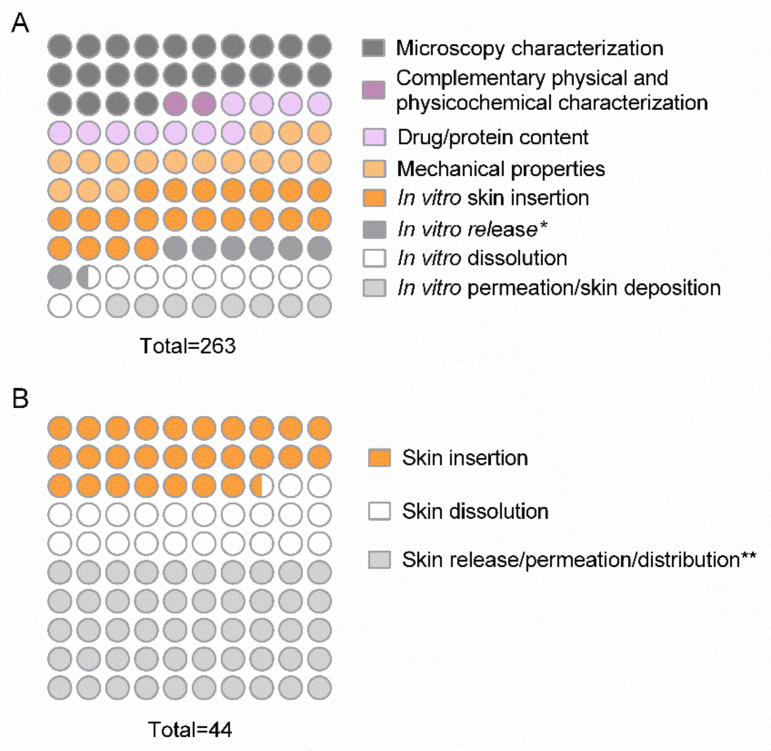
Dot plot of the tests employed to analyze polymeric MNs in association with nanostructured systems. (**A**) Microscopy characterization, complementary physical and physicochemical characterization (FTIR, TGA, DSC, and XRD), mechanical properties, and in vitro assays. (**B**) In vivo assays concerning skin insertion, skin release/permeation/deposition, and in situ dissolution. * It differs from the in vitro dissolution assay in that it quantifies the amount released. ** It differs from the in vivo skin dissolution assay in that it measures the amount released or permeated, including fluorescence measurement.

As shown in Figure 4, overall, the studies added up to 263 in vitro and microscopy assays and 44 in vivo assays. As described in the methodology, the assays applied for MN characterization were classified into eight subcategories for microscopy, physicochemical, mechanical properties, and in vitro methods and three subcategories for in vivo assays. After device preparation, 87.5% of the studies performed microscopic analysis, 76.4% evaluated in vitro skin insertion, 59.7% characterized the mechanical properties, 40.3% determined the content of the active substance carried in the device, and 38.9% elucidated the in vitro dissolution profile. Less frequently, 29.0% investigated in vitro skin permeation/deposition, 26.4% investigated the in vitro release profile, and only 6.9% of the studies applied other techniques for the characterization of the MN, such as thermogravimetric analysis (Figure 4A).

As previously mentioned, in vivo assays for the characterization of skin insertion, skin release/permeation, and skin dissolution capacity were employed less frequently than in vitro assays in the studies. Of these, skin insertion was evaluated in 12 studies, skin release/permeation in 22 studies, and dissolution in 10 studies (Figure 4B). Particularly in the category of in vivo trials, skin release, permeation, and deposition assays were addressed as synonyms in the evaluated studies.

#### 3.3.1. Microscopy and Complementary Physical and Physicochemical Characterization (FTIR, TGA, DSC, and XRD)

Verification of MN dimensions and shape by microscopy techniques was conducted in 63 studies. Out of these, 46 studies showed the microscopic aspect by scanning electron microscopy (SEM) and 4 by field emission scanning electron microscopy (FESEM). The second most applied technique was optical microscopy, reported in 22 studies as bright field microscopy, digital microscopy, and optical microscopy. The studies that evaluated the combination of fluorescence probes free in the polymer matrix or associated with the nanostructured system highlighted the morphological features by fluorescence microscopy (*n* = 14). In addition, 10 studies applied confocal laser scanning microscopy (CLSM technique) for characterization, and only 3 studies employed transmission electron microscopy (TEM). The distribution of the number of studies by characterization technique is detailed in Figure S2. In parallel, few studies characterized the devices with Fourier transform infrared spectroscopy (FTIR, *n* = 2) [55,64], X-ray diffraction (XRD, *n* = 2) [55,64], differential scanning calorimetry (DSC, *n* = 3) [55,64,65], and thermal gravimetric analysis (TGA, *n* = 2) [65,66] techniques. In comparison, FTIR, XRD, and DSC techniques were applied to nanostructured system characterization (Figure S1) in 11, 12, and 9 studies, respectively.

#### 3.3.2. Drug or Protein Content

Quantification of the substances, drugs, and proteins carried in the devices was performed in 29 studies. The sample preparation procedure consisted of the complete dissolution of the devices in distilled water, buffer, or mixtures with organic solvent and subsequent quantification [64,67]. For devices composed of at least two polymer layers, in which the nanostructured system was located only in the needle, whereas the baseplate was formed by an inert polymeric structure, the studies described the removal of the microneedles with a scalpel and only the needles were dissolved for analysis [33,34,38,51,52,68,69]. Images of the needles removed for testing and the remaining baseplate are shown in the article by Li et al. [68]. Additionally, Rojekar et al. described the procedures performed with solvent gradients (acetonitrile:water) to promote the precipitation of the polymers and PVA/PVP and, thus, allow the quantification of etravirine without interference from the MN matrix [70].

One study inferred the content of doxycycline, diethylcarbamazine, and albendazole in the needle tips by theoretical calculations based on the content present in the lyophilized nanoparticles and the MN density [22]. For density determination, a needle-free patch (formulation film) was initially prepared under the same conditions as the MNs. After drying, the film dimensions (width, thickness, and length) were measured to obtain the volume and the mass was verified, thus obtaining the density. The second step consisted in applying the Equation (1)) to determine the content. This requires knowing the dimensions of the needles (height and base diameter) as well as the content of the compounds in the nanoparticles and the mass of nanoparticles applied. Importantly, in the mentioned study, pyramidal MNs with square bases were obtained, so the equation should be modified according to the geometry for conical microneedles; for example, the appropriate equation is described in Equation (2).

(1) $$\mathrm{Drug}\;\mathrm{content}\;\left(\mathrm{mg}\right)\;\mathrm{in}\;\mathrm{the}\;\mathrm{MN}=N\;\times \frac{\left(h\;\times {\;a}^{2}\right)\times \;\rho \;\left[\mathrm{drug}\right]}{3}$$

where MN: microneedle; N: total number of needle tips; h: height of needle tip (mm); a: width of base tip (quadrangular) (mm); ρ: dry formulation film density (mg/mm^3^); [drug]: mg drug/mg lyophilized nanoparticle.

(2) $$\mathrm{Drug}\;\mathrm{content}\;\left(\mathrm{mg}\right)\;\mathrm{in}\;\mathrm{the}\;\mathrm{MN}=N\;\times \frac{\left(h\;\times {\;\pi r}^{2}\right)\times \;\rho \;\left[\mathrm{drug}\right]}{3}$$

where MN: microneedle; N: total number of needle tips; h: height of needle tip (mm); r: radius of base tip (mm); ρ: dry formulation film density (mg/mm^3^); [drug]: mg drug/mg lyophilized nanoparticle.

Similarly, two studies applied equations to determine the content of carvacrol and methotrexate in MN needles [41,69]. However, these differed from the theoretical method in that they prepared a needle-free patch containing the nanostructured substances and the density of the dry film was evaluated and the content of the substances in the film was measured by HPLC. Equation (3) describes the calculation to be applied after quantification.

As an alternative to the quantification method, some studies measured the uniformity of content in the needles, also called distribution, by adding dyes such as trypan blue [71], by including fluorescent substances in the MN, or by associating fluorescent probes to the nanostructured systems. In this way, the uniformity was observed by optical microscopy or fluorescence microscopy and CLSM [42,52,68].

(3) $$\mathrm{Drug}\;\mathrm{content}\;\left(\mathrm{mg}\right)\;\mathrm{in}\;\mathrm{the}\;\mathrm{MN}=N\;\times \left(\left(\mathrm{volume}\;\mathrm{formula}\right)\times \;\rho \;\left[\mathrm{drug}\right]\right)$$

where MN: microneedle; N: total number of needle tips; volume formula: according to needle shape; ρ: dry formulation film density (mg/mm^3^); [drug]: mg drug/mg film (experimentally determined).

#### 3.3.3. Mechanical Properties, In Vitro Assays

**Figure 5:**
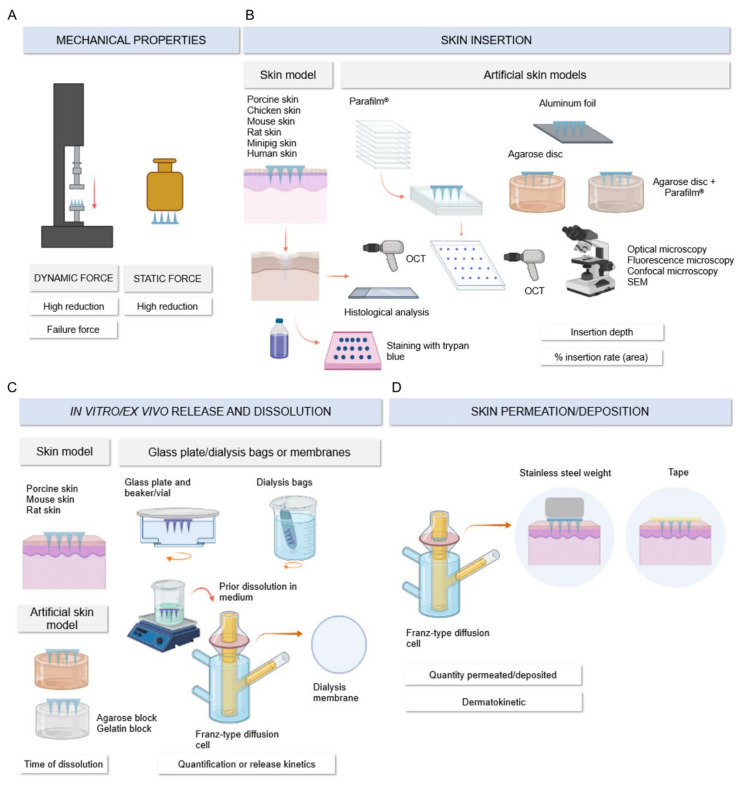
Graphic representation of the *in vitro* assays performed for the characterization of polymeric microneedles associated with nanostructured systems. (**A**) Mechanical properties: illustration of compression force by dynamic force with displacement-force test station (left) and by static force with the application of standard weights (right). (**B**) Skin insertion: skin model vs. artificial skin models (Parafilm^®^, aluminum foil, agarose disc, and agarose disc plus external Parafilm^®^ layer). (**C**) Release and dissolution assays: skin and artificial skin models (agarose and gelatin blocks) (left) and methods using specific glassware and dissolution medium (glass plate and beaker/vial, dialysis bags, or membranes) (right). (**D**) Skin permeation/deposition assay: representation of the technique with Franz-type diffusion cell and skin matrix. The white rectangles represent the results commonly obtained by the respective methods. OCT: optical coherence tomography.

Graphic representation of the main mechanical properties and in vitro methods employed in quality control is shown in Figure 5. Mechanical properties, denominated as compression force or failure force, were evaluated by dynamic force and static force techniques (Figure 5A). In the first technique, the device is fixed on a support using a double-sided tape, and then a standard perpendicular force is applied at a constant compression speed. The result is recorded as force vs. probe displacement. Graphical plotting of the results allows the determination of failure force, corresponding to the value at which the needles begin to buckle [20,47]. For this method, it is required to use equipment that allows this control, such as force-displacement testers and texturometers [72,73,74]. The experiment details applied in the mechanical characterization methods are presented in Table 1. The results revealed that the compression rate ranged from 0.008 to 1.19 mm/s, and the most frequent application force was 32 N. Looking into the static force technique, the applied weights ranged from 50 to 1000 g, and the holding time of each weight on the device varied from 1 to 5 min. Moreover, one study evaluated the mechanical properties using atomic force microscopy to obtain the force-displacement curves. In this case, a 10 mm sphere of SiO_2_ probe was applied with a 1 mN force at 500 nm/s [46].

The in vitro skin insertion assay, illustrated in Figure 5B, was the second most performed assay after microscopic characterization. The results demonstrated skin insertion measurement by skin models and artificial skin models; the last ones were performed by replacing animal skin with membranes and hydrogel matrices with thickness and elastic modulus similar to human skin. In skin models, the most applied skins were porcine and rodent skins, with less frequent application of chicken skin and human skin from surgical procedures. The skin insertion test parameters are detailed in Table 2. Regarding the mode of application, this can be conducted manually, with the aid of applicators previously calibrated to a single force value or with the equipment described in the mechanical characterization tests. Interestingly, some studies employed the same parameters in both tests, while others used the failure force value obtained to define the application force in the insertion test or to choose the most promising device in terms of mechanical resistance [38,41,51,56,70]. In addition to insertion force and mechanical strength, MN insertion capability is influenced by needle geometry and skin fixation support. Although 54 studies did not indicate needle tip diameter, 42 out of these confirmed insertion abilities by in vitro assays. In addition, some studies described skin fixation on a Styrofoam platform or dental wax to provide support for MN insertion [57,73,75,76].

**Table 1:** Parameters of the tests used to characterize the mechanical properties of microneedles in association with nanostructured systems.

Technique	Equipment	Applied Force (N)/Weight (g)	Compression Rate (mm/s)	Result Expression	Ref.
**Dynamic force**	CT3 texture	NI	NI	Compressive force-displacement curve	[66]
		NI	1.00	Compressive force-displacement curve	[77,78]
	Displacement-force test station (Model 921A)	NI	1.10	Compressive force-displacement curve	[72]
		10 N	0.008	Failure force	[60,79]
	DTS delaminator	10 N	0.05	Compressive force-displacement curve Failure force	[32]
	Force displacement tester (model 925)	NI	1.25	Compressive force-displacement curve Failure force	[42]
	Hounsfield universal mechanical testing machine	10 N	0.017	Compressive force-displacement curve	[23]
	Mechanical testing system (5943 MicroTester)	NI	0.50	Compressive force-displacement curve Failure force	[47,74,80]
		10 N	0.10	Compressive force-displacement curve	[81]
		NI	0.10	Compressive force-displacement curve Failure force	[82]
		50 N	0.50	Compressive force-displacement curve	[83]
	Side-shaking test stand (HCS-500) and a thrust meter (HF-50)	2, 4, 8, 12, 16, and 20 N	NI	Observations of the MN deformations with a digital camera	[84]
	Tensile load frame	NI	0.01	Compressive force-displacement curve	[85]
	Tensile machine (Instron)	NI	0.017	Failure force	[86]
	Tensile testing machine (MTS 30G)	10 N	0.10	Compressive force-displacement curve Failure force	[20,61]
	Texture analyzer (TA-XT2, Stable microsystems)	45 N	0.05	Percentage height reduction (digital microscopy)	[19]
		45 N, held for 30 s	NI	Percentage height reduction (digital microscopy)	[57,73,75]
		32 N, held for 30 s	1.19	Percentage height reduction (stereomicroscopy, digital microscopy)	[22,38,39,44,55,56,69,87]
		32 N, held for 30 s	0.50	Percentage height reduction (stereomicroscopy)	[41,51,70]
		NI	0.10	Compressive force-displacement curve Failure force Stereomicroscopy	[76]
		0.049 N	0.50	Failure force	[62]
		40 N	0.01	Failure force MN morphology (Scanning electronic microscopy)	[53]
	Texture analyzer (XT plus, Stable microsystems)	NI	1.00	Compressive force-displacement curve Failure force	[52]
	Universal testing machine (MARK-10)	NI	1.00	Compressive force-displacement curve	[68]
	Atomic force microscopy	1.0 mN, 10 mm SiO_2_ sphere probe	500 nm/s	The moduli of the needles were calculated from the force-displacement curves	[46]
	NI	NI	NI	Compressive force-displacement curve	[40]
**Static force**	Standard weight	50, 100, 200, and 500 g, held for 1 min	NA	Optical images of the MN deformation	[81]
	Standard weight	500 g, held for 5 min	NA	Optical images of the MN deformation	[47,74,80]
	Standard weight	100, 200, 500, and 1000 g, held for 5 min	NA	Optical images of the MN deformation and images by confocal microscopy	[88]

NI: not informed; NA: not applied.

**Table 2:** In vitro skin insertion assay parameters.

Model Category	Matrix	Insertion Force	Method of Mensuration	Observations	Ref.
**Skin model**	Chicken skin	Manual force	Histological analysis	-	[23]
	Human skin	Manual force, held for 30 s	CLSM	Skin from abdominal plastic surgeries	[53]
	Minipig skin	20.0 N	Histological analysis	Applicator: not informed	[18]
	Mouse skin	20.0 N	Staining with trypan blue	Equipment: Mechanical testing system (5943 MicroTester)	[83]
		Manual force, held for 5 min	Staining with trypan blue Histological analysis OCT	-	[35]
		NI	Staining with trypan blue Histological analysis	-	[89]
		NI, held for 3 min	Staining with trypan blue Histological analysis	-	[33]
	Mouse skin (Balb-c)	NI	Histological analysis	-	[85]
		NI	Force-displacement curve	Equipment: Texture analyzer, insertion speed 0.10 mm/s The skin was placed on Styrofoam block support	[76]
		NI	Histological analysis	-	[36]
	Porcine skin	1.0, 2.0, and 4.0 N	Staining with trypan blue	Insertion speed 0.5 mm/s The skin was placed on sheet of dental wax support topped with parafilm, and this set was fixed on a wooden block for support	[84]
		1.5 N	Staining with trypan blue Histological analysis Fluoresce microscopy	Homemade electric applicator	[71]
		8.0, 11.0, and 16.0 N	OCT	Spring-loaded applicator The skin was placed on sheet of dental wax support	[19]
		10.0 N	Digital microscopy Staining with Shandon™ Blue tissue marker dye Histological analysis	Spring-loaded applicator	[62]
		10.0 to 50.0 N	OCT	Equipment: TA-XT2 Texture Analyser, insertion speed 0.50 mm/s	[44]
		11.0 N	Histological analysis	Custom-made spring-loaded applicator; Insertion test in association with the permeation test (Franz-type diffusion cell)	[49]
		32.0 N, held for 30 s	OCT	Equipment: TA-XT2 Texture Analyser, insertion speed 1.19 mm/s or 0.50 mm/s	[22,38,39,41,51,56,70]
		Manual force (~1.5 N)	Fluorescence stereomicroscopy	-	[60,72,79]
		Manual force	OCT	The skin was placed on sheet of dental wax support	[57,73,75]
		Manual force	Stereomicroscopy Histological analysis	Evaluation of single, double, and triple insertion	[45]
		Manual force, held for 5 min	Staining with trypan blue Histological analysis	-	
		NI	Staining with trypan blue Histological analysis	-	[67]
		NI	Digital images of skin MN prepared with brilliant blue dye >> penetration efficacy	-	[64]
		NI	Staining with trypan blue	Equipment: CT3 texture, insertion speed 20.0 mm/s	[77,78]
		NI	SEM Fluorescence microscopy	-	[50]
	Rat skin	Manual force (~5 N)	Staining with trypan blue	-	[88]
		NI, held for 1 min	Staining with trypan blue Histological analysis CLSM OCT	-	[43]
	Rat skin (Sprague–Dawley)	NI	CLSM	-	[47]
		NI	Histological analysis CLSM	-	[74,80,82]
		NI	Staining with trypan blue Histological analysis	-	[90]
		NI, MN held for 3 min	Histological analysis Staining with trypan blue	-	[34,68,91]
		15 N, held for 1 min	Staining with trypan blue Histological analysis CLSM	-	[92]
	Origin not described	0.08 N	Fracture force	Equipment: CT3 texture, insertion speed 0.50 mm/s Additional analyses of bioadhesion and post-wetting bioadhesion	[54]
**Artificial skin model**	Agarose disc (3% *w*/*v*)	Manual force, held for 1 min	Fluorescence microscopy	-	[42]
	Agarose disc covered with a Parafilm^®^ layer (2% *w*/*v* agarose disc, thickness: 6 mm, Parafilm^®^ layer: 127 µm)	NI	SEM	Equipment: TA.XT plus texture analyzer, insertion speed 1.00 mm/s	[53]
	Aluminum foil	Manual force	Observation of the holes in the aluminum foil	-	[88]
	Gelatin hydrogel (5% *w*/*v*)	NI	Optical microscopy	-	[65]
	Parafilm^®^ (8 layers, ~1 mm)	10.0 to 50.0 N	OCT	Equipment: TA-XT2 Texture Analyser, insertion speed 0.50 mm/s	[44]
		32.0 N, held for 30 s	Digital microscopy (number of holes/layer) OCT	Equipment: TA-XT2 Texture Analyser, insertion speed 1.19 mm/s or 0.50 mm/s	[22,38,39,41,51,55,56,69,70]
		Manual force, held for 5 min	SEM	-	[93]
		Manual force (~30 N) vs. 50 N	OCT	Equipment: Instron universal testing instrument model 5567, insertion speed 0.50 mm/s	[48]
	Parafilm^®^ (10 layers, ~1 mm)	30 N, held for 5 min	Digital microscopy (number of holes/layer)	Equipment: XT plus Texture Analyzer, insertion speed 1 mm/s	[52]

SEM: scanning electron microscopy; CLSM: confocal laser scanning microscopy; OCT: optical coherence tomography; NI: not informed; MN: microneedle.

Alternatively, skin insertion and in vitro dissolution assays were performed simultaneously on skin samples. For this purpose, the MNs were inserted and removed after pre-established times to investigate the dissolution profile. After the removal of the MNs, they were analyzed for length reduction, and the skin sample was stained with trypan blue solution and analyzed histologically. Through the trypan blue staining, it was possible to observe the holes marked in blue, referring to the insertion, and to calculate the insertion percentage, adopting as a reference value (100%) the total number of needles present in the device [71,90]. Through the histological analysis technique, by staining the histological sections with hematoxylin and eosin, the depth of the insertion was observed, and it was certified that the MN reached the epidermis, piercing the stratum corneum [43,45]. A third technique for monitoring the insertion depth applied in the studies was optical coherence tomography (OCT), as represented in Figure 5B. This technique is widely used in preclinical studies and medical sciences because it is non-invasive and presents images without the need for specific sample preparation, such as the preparation required for histological analysis [94]. By observing the insertion area, the depth of the insertions can be easily measured as well as the thicknesses of the skin layers by the cross-sectional images [19,56,57].

In accordance with the efforts of the scientific community for the validation and application of alternative methods to the use of animals, it was notable that a considerable number of studies applied artificial skin methods to evaluate the insertion. For skin replacement, the Parafilm^®^ model was employed, in which eight layers were combined, totaling a thickness of around 1 mm, and the insertion of MN was measured by the depth of penetration and by the number of holes per layer, easily observable using a digital camera or an optical microscope [51,56]. Other more sophisticated techniques such as OCT were also used to measure the depth of insertion [22,44]. Since this method was validated in 2014 by Larrañeta et al. [95], this model was only cited in the included studies from 2017 onwards. The other alternative methods to using skin were mentioned from 2019 (gelatin) and 2020 (agarose and aluminum foil) [65,88].

Given that 71 studies have developed dissolving MNs, one of the essential tests for monitoring this quality is in vitro dissolution since the release rate of the nanostructured system from the polymer matrix is influenced by the dissolution time. According to the extracted data and as illustrated in Figure 5C, the evaluation of in vitro dissolution was performed in models using skin, artificial skin, or glassware and an aqueous dissolution medium. The models are detailed in Table 3.

As presented, the most employed model refers to the use of skin, especially porcine skin, followed by the in vitro model, with the application of gelatin blocks (5% and 35% *w*/*v*) to simulate the skin. The insertion force was not mentioned in most studies, and some reported application by manual pressure. To avoid the slippage of MN arrays from the skin, some studies placed a standard stainless-steel weight (5.0–13.0 g) on the device after removing manual pressure until the required analysis time. Most studies evaluated dissolution at 37 °C to simulate body temperature. The assay time varied between 30 s and 2 h, and the dissolution of the NMs was measured by the percentage of needle length reduction or by morphology modification compared to the uninserted MN. For this, the most used techniques were light microscopy and CLSM.

Adaptations of the dissolution assay were highlighted in some studies to simulate specific conditions of the proof of concept proposed in the study. To prove the dissolution and glucose-dependent system, Jiang et al., Tong et al., and Xu et al. used skin from SD rats in normal and hyperglycemic conditions [74,80,82]. Permana et al. simulated porcine skin affected by bacterial biofilm to confirm the release ability of doxycycline hyclate nanoparticles developed for antibacterial action [38].

Concerning the in vitro release assay, the experimental details are reported in Table 3, and the graphical illustration of the techniques is also shown in Figure 5C. Different from dissolution, which evaluates the morphological aspects of MNs, this assay aims to quantify the portion of active substance released over time. For its execution, a glass plate/beaker and vial, a bag, and dialysis membranes were employed; a single study applied USP Dissolution Apparatus 5 (paddle over disk). The former corresponds to a simplified methodology, in which the MN is fixed on a support such as a plate or the wall of a beaker and exposed to the dissolution medium under stirring. At pre-set times, an aliquot of the medium is collected, taking care to replace the removed volume with fresh medium, and the amount present in the aliquot is quantified. The technique for quantification depends on the characteristics of the analyte and the available laboratory infrastructure. It is important to emphasize that the methods chosen must be previously validated and appropriate to indicate the selectivity of the substance of interest against the polymeric materials used.

In reference to the methods that apply bags and dialysis membranes, these allow the separation of the free substance released from the nanostructured system as well as the retention of the dissolved polymer inside the bag or on the semipermeable membrane with a molecular weight cut-off [52,63,77]. Less frequently, agarose gel and skin model methods were employed. The latter determined the amount released indirectly. After 3 min of MN skin insertion, the remaining MNs were dissolved in distilled water and quantified using UV spectrophotometry. The released quantity was calculated as the difference between the total quantity and the remaining quantity [33].

Looking at the assay parameters, medium compositions that varied according to substance specificities and different pH values were employed. Some studies explored pH values representative of blood pH (pH 7.4) and skin pH (pH 5.5). Similar to the dissolution assay, one study simulated hyperglycemic conditions in order to check whether the developed system was sensitive to different glucose concentrations [46]. However, temperature also ranged from 32 to 37 °C, representing normal skin surface temperature and internal temperature. Regarding rotations, they ranged from 50 to 500 rpm, while the analysis time ranged from 20 s to 24 h. Importantly, the medium and rotations applied in the release assays must ensure the sink condition during run time to allow the release gradient.

**Table 3:** In vitro dissolution and release assays parameters.

Dissolution Model/Apparatus	Matrix/Medium	Temp.		Time of Insertion	Insertion Force	Dissolution Measurement	Ref.
Skin model	Porcine skin	37 °C NI		0.5–60 min NI	Manual force + weight 5.0 g Manual force + weight 13.0 g Manual force NI	Digital microscopy Fluorescence microscopy OCT Optical microscopy SEM Stereoscopic microscopy	[22,38,41,50,51,55,58,62,69,71,75,77,78,79,87,88]
	Mouse skin (Balb-c)	NI		30–120 min	NI	CLSM	[76]
	Rat skin (Sprague–Dawley)	NI		0.5–30 min	NI	Bright field microscopy CLSM 3D-CLSM SEM	[47,90,91] Health and diabetics rats [74,80,82]
Glass plate/beaker and vial	PBS	37 °C NI		<0.5–10 min	NA	Confocal microscopy Optical microscopy	[59,93]
	Water	NI		0.33–1 min	NA	Optical microscopy	[66]
Gelatin block	Gelatin block (5% *w*/*v*)	NI		0.33–1 min	NI	Optical microscopy	[65]
	Gelatin block (35% *w*/*v*)	NI		10 min	NI	Digital microscopy	[35]
**Release Assay/** **Apparatus**	**Matrix/Medium**	**rpm**	**Temp.**	**Time Assay Range**	**Insertion Force**	**Quantification Methods**	**Ref.**
Agarose gel	1% *w*/*v* agarose gel containing different concentrations of glucose	NA	NI	10–180 min	NI	Fluorescence stereomicroscopy QIAquick gel extraction kit (ELISA)	[60]
Dialysis bags	PBS (pH 7.4)	NI	NI	4–72 h	NA	HPLC	[63]	
	30% (*v*/*v*) PEG 400 in saline	250	32 ± 1 °C	1–24 h	NA	HPLC	[52]
Dialysis membranes (Franz-type diffusion cell)	30 % *v*/*v* ethanol solution in distilled water	NI	NI	10–1440 min	NA	HPLC	[77,78]
Glass plate/beaker and vial	PBS (pH 5.5)	500	37 °C	1–120 min	NA	Fluorescence spectroscopy	[50]
	PBS (pH 7.4)	50–100 NI	37 °C	1–1440 min	NA	Fluorescence spectroscopy CLSM UV–vis spectroscopy	[23,53,85,93]
	PBS (pH 7.5)	NI	37 °C	<3 days	NA	Fluorescence spectroscopy	[58]
	Distilled water PBS (pH 6.8) containing 1% tween 80	200	37 °C	15–1440 min	NA	HPLC	[64]
	Glucose solutions at different concentrations (5.5, 11.1, and 22.2 mM) Saline	NI	37 °C	10–240 min	NA	Bradford protein assay kit	[46]
USP dissolution apparatus 5 (Paddle-over disc method)	PBS (pH 5.5)	NI	37.5 °C	5–1980 min	NA	UV–vis spectroscopy	[54]
NI	DPBS with or without collagenase (2 U/mL)	NI	NI	<120 h	NA	Picogreen kit (quantification of DNA release)	[83]
	PBS	80	37 °C	1–60 min	NA	Picogreen kit (quantification of DNA release) UV spectrophotometry	[96]
	PBS + glutathione	NI	37 °C	4–72 h	NA	Standard bicinchoninic acid assay (BCA) HPLC	[40]
	Water	NI	NI	20–60 s	NA	UV spectrophotometry	[66]
Skin model	Mouse skin	NA	NI	3 min	NI	UV spectrophotometry	[33]

Temp: temperature; rpm: rotation per minute; SEM: scanning electron microscopy; CLSM: confocal laser scanning microscopy; OCT: optical coherence tomography; NA: not applicable; NI: not informed; PBS: phosphate buffer saline.

**Figure 6:**
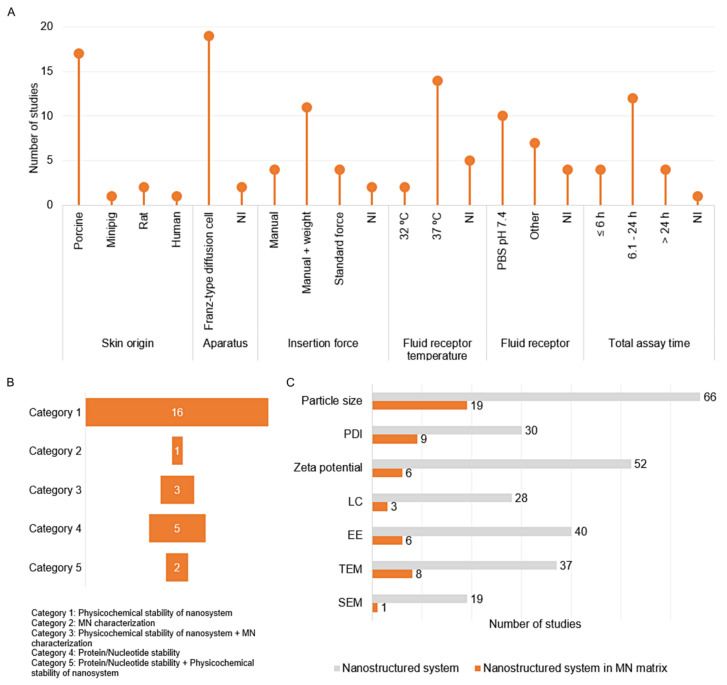
General parameters of the *in vitro* permeation assay by a number of studies (**A**). Characteristics of the stability tests by category (**B**), and comparative graph of the analysis of the nanostructured systems before and after insertion into the polymeric matrix of the microneedle (**C**). NI: not informed; PBS: phosphate buffer saline; PDI: polydispersity index; LC: loading capacity; EE: encapsulation/entrapment efficiency; TEM: transmission electron microscopy; SEM: scanning electron microscopy.

In the category of in vitro permeation assays, assays that evaluated skin retention/deposition as well as the permeation of compounds through all layers of the skin to reach the receptor fluid were grouped together. For nomenclature purposes, this assay will be referred to in the text as in vitro permeation. Notably, these parameters were evaluated in the studies using animal or human skin (*n* = 21), mainly porcine skin (*n* = 17), and no alternative methods were proposed for this evaluation. As illustrated in Figure 5D, the skin samples in most assays (*n* = 19) were fixed in Franz-type diffusion cells and conditioned with PBS pH 7.4 (*n* = 10) for at least 30 min prior to insertion of MNs into the skin. Unlike the in vitro dissolution assay, insertion force was described in most studies that evaluated permeation (*n* = 19), and, to avoid slippage of MN arrays from the skin during the assay, 11 studies employed the technique of overlaying the MN with a standard stainless-steel weight. Alternatively, the device was fixed with tape. The most usual temperature for the receptor fluid receptor was 37 °C, and some studies reported keeping the receptor fluid at 37 °C, while the skin surface was kept at 32 °C, similar to physiological conditions. The agitation speed of the receptor fluid varied from 100 to 600 rpm. Regarding the assay time, the in vitro permeation assay demonstrated longer sample collection intervals, owing to the construction of the dermatokinetic profiles of the compounds [38,41,69,88]. The graphical distribution of the variables described by a number of studies is shown in Figure 6A.

Out of 21 studies, 15 studies evaluated the developed device against controls. The chosen control groups varied among the studies; some applied needle-free patches produced with the same materials, i.e., polymeric films containing the nanostructures [22,49,55,56,87]. Some studies employed micro-needles containing the substances in their free form, i.e., without the presence of nanostructured systems [41,54,69,77]. Additionally, one study evaluated the modification of the polymeric composition on the permeation profile by applying MNs of hyaluronic acid and micelles without the presence of CMC [88]. The other studies applied the nanostructured systems in suspension or solution on the skin [51,77,87], and one study applied a suspension of the nanostructured system after the microporation of the skin with solid MNs.

At the end of the test time, the quantity of active substances permeated or retained in the skin layers was mostly measured by HPLC (*n* = 16) and spectroscopic or spectrophotometric techniques (*n* = 5). For the studies that evaluated dermatokinetic profiles, after quantification, the data were analyzed with PkSolver software (one-compartment model) [22,38,87]. Additionally, for the separation of the skin layers, two main techniques were employed. The first consisted of exposing the skin to a 60 °C bath for 2 to 3 min, followed by the removal of the epidermis with the aid of tweezers [22,44,69]. In the second, the skin sample was fixed in a material suitable for histological sectioning, and then the substance was quantified from the skin sections [22,41].

##### External Stimulus Application

In complement to the device formed by the combination of dissolvable polymers and a nanostructured system, the drug and protein release process can be promoted through external stimuli such as iontophoresis and magnetism fields as well as be applied to potentiate the therapy as near-infrared-light (NIR). Among the studies included in this review, only eight described the use of external stimuli, of which five studies corresponded to NIR [33,34,35,36,43], one to photodynamic therapy [85], one to iontophoresis [23], and one to magnetic fields [66]. The tests performed to evaluate the effects of these stimuli on the nanostructured systems and devices are presented in Table S2. Importantly, most studies that have applied laser radiation have measured the photothermal effect of the nanostructured system alone in vitro methods, evaluating this effect on the device (MN + nanosystem) only in in vivo models.

##### Stability Assays

The characteristics of the stability studies are shown in Figure 6B,C. According to the tests and storage parameters of the devices, the stability tests were divided into five main categories: (1) studies that evaluated the maintenance of the characteristics of the nanostructured system after insertion into the MN; (2) characterization of the MN; (3) physicochemical characterization of the inserted nanostructured systems and the MN characteristics; (4) protein/nucleotide stability after insertion into the MN; (5) physicochemical characterization of the nanostructured systems and protein/nucleotide stability after insertion into the MN. In all, 27 out of 72 studies measured one of the categories, and of these, only three followed the International Conference on Harmonization (ICH) guideline for stability studies regarding storage conditions: (a) long-term stability (25 ± 2 °C/60% RH ± 5%), (b) accelerate stability (40 ± 2 °C/75% RH ± 5%) [64], and (c) short-term stability (30 ± 2 °C/60% RH ± 5%) [77,78].

As shown in Figure 6C, in comparison to the analyses of the nanostructured systems after their preparation, the stability studies investigated the maintenance of particle size (*n* = 19) and, less frequently, the polydispersity index (*n* = 9) and morphological appearance by TEM (*n* = 8) after insertion into the MN. The maintenance of MN mechanical characteristics was measured in 3 studies [70,77,78]. Considering all the stability studies, it is important to note that 40.74% reported the follow-up times after MN preparation, and 29.63% reported the storage temperature of the MN.

#### 3.3.4. In Vivo Assays

The in vivo trials associated with the quality of MNs to ensure transcutaneous release fall into three main groups, as shown in Figure S3. The parameters were grouped into characteristics of the animals used (such as species/breeding line, age, sex, and sample number), parameters of the assays, and methods for measuring the results. For all three assays, it was evident that rats and mice were used equally, and there was no clear relationship between the type of assay and the age or sex of the animals. A significant number of studies did not indicate the number of animals employed in each trial. Furthermore, the insertion site of MNs showed the following order of preference: dorsum skin (back) > ear skin > abdominal skin. Compared to in vitro insertion assays, the insertion force applied in vivo was described to a lesser extent (*n* = 2). The in vitro dissolution time was less than 30 min and the follow-up time for skin release and permeation was similar to the in vitro studies with maximums of 24 and 48 h.

## 4. Discussion

### 4.1. Summary of Evidence and Characteristics of MNs Associated with Nanostructured Systems

The geographical distribution of the articles (Figure 2B) followed the market trends presented in market analysis reports, which show the participation of South America in the commercialization of solid and hollow MNs, substantiating the absence of studies on polymeric MNs in this region. On the other hand, continents such as East Asia, Europe, and North America, which had the highest number of publications included in this review, present market trends aligned with the development of polymeric MNs with the application of biodegradable and dissolvable polymers [16].

With respect to the polymers employed in the preparation of MNs, a vast chemical variety was observed, with emphasis on the use of PVP, PVA, and the PVP/PVA blend. Both polymers demonstrate characteristics such as biocompatibility and mechanical strength compatible with the application of the device, as well as fast in situ dissolution [41,47,51,97,98]. Another advantage associated with these polymers corresponds to the range of molecular weights that are marketed, allowing combinations with different dissolution rates and mechanical strength [57,74,76,84]. When evaluating the biodistribution and pharmacokinetic profiles of polymers such as PVA and PVP intravenously, some studies have shown that the half-life decreases and glomerular filtration is favored when low molecular weight PVA and PVP are administered. For example, PVA elimination is favored for polymers with molecular weight <30 kDa and for PVP of <25–50 kDa [97,99,100]. The wide range of molecular weights employed in the constitution of polymeric MNs in association with nanostructured systems was remarkable, but most applied PVA from 6 to 23 kDa and PVP from 31 to 50 kDa. Moreover, a significant number of studies applied only PVP of higher molecular weight in the baseplate (MW 360 kDa), keeping only the needle tips as a reservoir of nanostructured active compounds. This is a smart strategy since only the needle tips will encounter the deeper layers of the skin and the baseplate is removed intact after administration [21,22,44]. In contrast to the dissolving polymers, only one study employed hydrogel-forming polymers, evidencing that the association of hydrogel-forming MNs and nanostructured systems is less frequent than dissolving ones [48].

The association of nanostructured systems in TDDS such as MNs allows for one-step administration and control of the release rate by modifications of the polymer matrix (dissolution) and the dissociation rate of the loaded substance in the nanostructured system [54,77,78]. As evidenced in the step of selection of studies, in the first studies, the administration was performed in two steps, in which the skin was previously microporated by a solid MN and then the nanostructured system was administered, combined with semisolid formulations [101,102,103]. In addition to the simplicity of administration, the devices can be designed for controlled release to treat a particular pathophysiological condition, as demonstrated in the studies that designed the release for hyperglycemic conditions [20,47].

Beyond the control of hyperglycemia, most systems were designed for the treatment of chronic diseases and superficial tumors and mainly for transcutaneous immunization, owing to the cutaneous immune system [40,43,59,84,104]. The difficulties related to the penetration and permeation of compounds into tumor tissues and bacterial biofilms could be circumvented with needles projections [37,38,43]. In the case of tumor treatment, the systems can be designed to associate thermo-sensitive substances with chemotherapy, allowing the application of external stimuli (photothermal therapy) and, consequently, potentiating the treatment [35,36].

Regarding the types of associated systems, lipid, metallic, inorganic, and polymeric systems were retrieved in the search. Most studies developed polymeric nanoparticles due to their compatibility with the polymeric matrix, whereas simpler systems in terms of composition, such as nanoemulsions, were not retrieved in the results. Among the lipid systems, liposomes represented the largest fraction of these systems as they exhibit considerable biocompatibility in vivo and compounds of protein and nucleotide origin are easily associated with liposomal systems [38,71,78,105]. 

As for the method of obtaining the devices, after preparing the nanostructured systems by different techniques, the MN was mostly made using the micromolding method. The preference for micromolding is due to the versatility of this technique, which allows the obtaining of MNs with different shapes and sizes as well as the reuse of the female mold, usually composed of PDMS. As previously presented, most of the studies developed pyramidal and conical MNs; both shapes present a favorable geometry for cutaneous insertion. Moreover, the studies indicate that the pyramidal shape confers greater drug loading when compared to conical needles of the same height and base width. The influence of geometric aspects on the ability of cutaneous insertion has been investigated in previous studies, which showed that the geometry directly influences the shape and length of the conduits in the skin and that the insertion is favored in pyramidal needles with a triangular or square base, with less intensity for hexagonal bases [106,107]. The conical format was one of the first to be explored in the constitution of MNs. For solid MN, it is important to highlight that some studies evaluated the viability of production of PDMS female molds from solid MNs commercialized for aesthetic microneedling (Dermastamp^®^) and even from tattoo needles [77,107]. Parallel to this technique of obtaining the base male mold for the PDMS mold, two papers demonstrated the possibility of using 3D printing, an emerging technique applied to pharmaceutical devices and forms [53,66].

### 4.2. Assays Employed in MN Analysis

In relation to the characterizations of the dimensions and shapes of the MNs, these were verified by microscopic techniques, mainly by SEM. As for the characterization of the isolated nanostructured systems, the most used technique was TEM [40,93,108]. These differences were attributed to the specificities of the materials used. Studies that applied fluorescent substances and probes employed fluorescence microscopy and CLSM as complementary techniques. In these studies, the addition of fluorescent probes allowed the observation of particle distribution in the needles as well as associated fluorescence techniques for the visualization of cutaneous release and permeation, both in vitro and in vivo techniques. Some studies also justified the choice of probes by the similarity of molecular weight and lipophilicity with the active compound [61,69,79,109]. This attention to choice by physicochemical similarity is important since the profile of skin permeation and release is associated with these variables.

Significant differences were also observed between the number of studies that characterized the nanostructured systems in terms of particle size, PDI, and zeta potential, before and after mixing in the MN polymer matrix. One possible cause for this difference relates to analytical limitations, as some studies highlighted that the polymer could interfere with the analysis [63,70]. Nevertheless, only 40.3% of the studies described the determination of the content of the substances in the MN.

The mechanical properties of the devices, represented by the compressive strength, were evaluated by dynamic and static strength techniques. The former represented the largest fraction of the studies, mainly by informing the failure force value, which predicts the subsequent capability of skin insertion. In general, the compressibility tests are extremely useful in understanding the effects of the nanostructured system on mechanical strength. When comparing with MNs composed of polymer only, some studies identified differences in the failure force value. It cannot be understood as a rule; however, inorganic (mesoporous nanoparticles) and metallic nanostructured systems may provide higher mechanical strength to the final device, while lipid and liposomal systems may reduce this value [23,47]. Another advantage of the mechanical characterization technique is the possibility of adjusting the formulations before conducting more complex and costly studies such as skin release and permeation [69].

Regarding skin insertion assays, assay features in terms of application mode and force employed were described more frequently in the in vitro studies than in the in vivo studies. Furthermore, it was verified that the studies assigned manual force values of 1.5 to 30 N [48,79,88]. In an attempt to standardize manual strength, Larrateña et al. measured the manual force of 20 volunteers (10 males and 10 females) by pressing their thumbs on the TA.XTPlus Texture Analyser (Stable Micro Systems) platform and holding the pressure for 30 s [95]. Afterwards, the results were evaluated in terms of average, maximum, and minimum force during the interval. In this study, the average contracted force was 20 N, with minimal differences between males and females. Additionally, when mimicking the manual application of MNs by the volunteers to porcine neonatal skin, the authors showed a greater statistical difference in insertion for those who employed a force of less than 10 N. Although these results contrast the manual forces of 1.5 and 5 N employed by other authors, these confirmed the insertion by fluorescence microscopy and OCT and that insertion capacity is not only a result of the force employed but also of the geometry [106,110].

The term dissolution was used in most studies as a reference to the in vitro assay to assess the ability or time for morphological changes to occur in the structure of NM after insertion into a skin matrix, simulated skin matrix, or even a liquid medium. Although the term dissolution is used in pharmaceutical sciences to refer to the experiment by which the rate and extent that a compound forms a solution is determined, the result is usually expressed as a percentage of the amount dissolved over time relative to the total amount. In addition, official compendia recommend the use of specific apparatus and dissolution media for conducting the dissolution assay, such as USP Apparatus 1 (basket) and 2 (paddle), according to standardized conditions [111]. It was observed that the studies referred to the quantification assays after the dissolution of the polymeric matrix as release assays, and the conditions were variable among the studies regarding equipment and media. Therefore, for the purpose of grouping the results, the nomenclature in this review was standardized in the same way as in the studies.

In vitro skin insertion and permeation assays, in general, demonstrated the majority use of porcine skin compared to other animal origins. Several studies have evidenced the physiological similarity of porcine skin to human skin, especially in the thickness of the epidermis and lipid composition, thus conferring permeability similar to that of human skin [112,113,114,115]. However, for the use of porcine skin, some precautions should be taken during the preparation of the skin matrices, such as thickness standardization (<1 mm). Likewise, for skin samples stored by freezing, the samples should be rehydrated with PBS pH 7.4 solution for at least 30 min before conducting the assays [81,116].

### 4.3. Limitations

While conducting the review, some limitations were evident, such as a lack of standardization of the nomenclatures adopted in relation to quality testing in the articles and difficulty of access to some variables. Probably due to character limitations or specifications of the guidelines for authors, they chose to emphasize the results of their research and the novelty of state of the art, reducing the information presented regarding methods and analysis parameters. Therefore, one of the limitations in conducting this review was the methods partially described in the original articles or the corresponding Supplementary Materials.

### 4.4. Future Possibilities and Potential of Polymeric MNs

From the results raised in this review, some future possibilities are pointed out:

- Development of specific alternative methods for the evaluation of TDDS as well as the validation of in vitro methods to characterize the dissolution and release profiles of substances from MNs containing nanosystems; development of specific equipment and apparatus to assess these parameters more reliably against physiological skin conditions;
- Evaluation of aspects that directly or indirectly impact the product profile, for example, the mechanical force required for the insertion of the device into the skin. One way to evaluate this parameter, indicative of future self-administration success rate, is to evaluate the mechanical characteristics and in vitro or in vivo skin insertion. Since different individuals have distinct hand strengths, the validation and standardization of these assays is critical to understanding and predicting the consequences of this variability;
- The standardization of quality methods will boost the growth of polymeric MNs in the market as well as allow the evaluation of systems in line with the trend of personalized medicine, especially for the treatment of chronic diseases and associated comorbidities;
- The association of nanostructured systems and polymeric matrices for the transcutaneous administration of substances will be enhanced if different strategies that modulate drug release are combined (different systems, different polymeric layers, or where the combination of substances in free form and those associated with nanosystems are introduced in the same matrix). However, control methods will have to be developed to characterize these systems;
- The lack of investment in stability studies that prove the maintenance of nanometric characteristics after inclusion in the polymeric matrix may represent a breakpoint in the process of scaling up from the bench to market.

## 5. Conclusions

The described work is the first demonstration, to our knowledge, of a review that compiles the tests employed in the quality control of polymeric microneedles prepared in association with nanostructured systems. From the results, it was observed that the most used tests for quality assessment are microscopic analysis, characterization of mechanical properties, skin insertion capacity, dissolution and release profile, and skin permeation/retention. However, there was a higher percentage of non-animal methods for the analysis of skin insertion and dissolution in the more recent studies. Furthermore, the application of alternative methods to animals to verify insertion and dissolution was more frequent in studies published from 2017 onwards, in line with the recent validation of alternative methods. Finally, the compiled data ensure the requirement for standardization of testing and its execution to narrow the proof-of-concept and market spheres of polymeric microneedles associated with nanostructured systems and support the establishment of regulatory guidelines for these devices.

## Supplementary Materials

The following are available online at https://www.mdpi.com/article/10.3390/pharmaceutics13101601/s1, Table S1: Detailed search strategy applied to PubMed, Embase, and Web of Science databases, Table S2: Description of the assays applied to evaluate external stimuli in the release and therapeutic effect of nanostructured systems associated with dissolving microneedles, Figure S1: Characterization tests of nanostructured systems prior to inclusion into the polymeric matrix of the microneedle by a number of studies included in the review, Figure S2: Characterization tests of the MN device by a number of studies included in the review, Figure S3: General parameters of in vivo skin insertion assay.

## Abbreviations

3D-CLSM: three-dimensional confocal laser scanning microscopy, CAGR: compound annual growth rate, CLSM: confocal laser scanning microscopy, CMC: sodium carboxymethylcellulose, DSC: differential scanning calorimetry, FESEM: field-emission scanning electron microscopy, FTIR: Fourier transform infrared spectroscopy, ICH: International Conference on Harmonization, m-HA: methacrylated hyaluronic acid, MN: microneedles, NIR: near-infrared-light; PLGA: poly(lactide-*co*-glycolide), PMVE/MA: copolymer of methylvinyl ether and maleic anhydride, PVP: polyvinylpyrrolidone, PVP/PVA: polyvinylpyrrolidone/poly (vinyl alcohol), SEM: scanning electron microscopy, TDDS: transdermal drug delivery system, TGA: thermal gravimetric analysis, XRD/XRPD: X-ray diffraction/X-ray powder diffraction.
