# Supplementary material for: Dissolving Microneedles Developed in Association with Nanosystems: A Scoping Review on the Quality Parameters of These Emerging Systems for Drug or Protein Transdermal Delivery
Source: Pharmaceutics. 2021 Oct 2;13(10):1601. doi: 10.3390/pharmaceutics13101601 (PMC8538119; doi:10.3390/pharmaceutics13101601)
Supplement: Supplementary file 1 [file pharmaceutics-13-01601-s001.zip › pharmaceutics-1365133-supplementary.pdf]

SUPPLEMENTARY MATERIAL

Polymeric microneedles developed in association with nanosystems: A scoping review on the quality parameters of these emerging systems for drug or protein transdermal delivery

Patrícia Weimer[1]; Rochele Cassanta Rossi[2]; Letícia Scherer Koester[1*]

Affiliation

[1] Programa de Pós-Graduação em Ciências Farmacêuticas, Faculdade de Farmácia, Universidade Federal do Rio Grande do Sul (UFRGS), Av. Ipiranga 2752, Santa Cecília, Zip code 90610-000, Porto Alegre, Rio Grande do Sul, Brazil.

[2] Programa de Pós-Graduação em Nutrição e Alimentos, Universidade do Vale do Rio dos Sinos (UNISINOS), Av. Unisinos, 950, Cristo Rei, Zip code 93022-000, São Leopoldo, Rio Grande do Sul, Brazil.

*** Correspondence**

Prof. Dr. L. S. Koester. E-mail address: leticia.koester@ufrgs.br | Address: Av. Ipiranga 2752, Santa Cecília, Zip code 90610-000, Porto Alegre, Rio Grande do Sul, Brazil | Phone.: +55 51 33085278; Fax: +55 51 33085437.

TABLES

Table S1 - Detailed search strategy applied to PubMed, Embase, and Web of Science databases

Database	Search strategy
MEDLINE	(((Microneedle*[Tiab] OR "microneedle array*"[Tiab] OR "microarray patch*"[Tiab] OR "microneedle patch"[Tiab] OR "dissolving microneedle*"[Tiab] OR "dissolving microneedle array*"[Tiab] OR "hydrogel forming microneedle*"[Tiab]) NOT ("solid microneedle*"[Tiab] OR "solid microarray*"[Tiab] OR "coat* microneedle*"[Tiab] OR "microneedle coat*"[Tiab] OR "coat* microarray*"[Tiab] OR "hollow microneedle*"[Tiab])) AND (Nanoparticle*[Tiab] OR Nanoparticles[MeSh] OR Nanostructures[MeSh] OR nanocapsule*[Tiab] OR nanocapsules[MeSh] OR nanoemulsion[Tiab] OR nanoconjugate*[Tiab] OR nanoconjugates[MeSh] OR nanocrystal*[Tiab] OR "metal nanoparticle*"[Tiab] OR "metal nanoparticle"[MeSh] OR "polymer* nanoparticle"*[Tiab] OR "polymer* nanocapsule*"[Tiab] OR nano*spher*[Tiab] OR lipo*some*[Tiab] OR liposomes[MeSh] OR nio*some*[Tiab] OR nanolip*[Tiab] OR "nanolip* particle*"[Tiab] OR "polymer* micelles"[Tiab] OR "solid lipid* nanoparticle*" [Tiab] OR nanosystem*[Tiab] OR "nano*structur* lipid* carrier*"[Tiab] OR "nano*structur* carrier*"[Tiab] OR nanocarrier*[Tiab] OR nanovesicle*[Tiab])) AND ("transdermal delivery"[Tiab] OR "transdermal patch"[MesH] OR "Administration, Cutaneous"[MeSh] OR "Administration, Transcutaneous"[MeSh] OR transcutaneous[Tiab] OR "transdermal drug delivery"[Tiab] OR "drug delivery"[Tiab] OR "drug delivery systems"[Mesh] OR "delivery system*"[Tiab] OR "drug therapy"[Tiab] OR "drug therapy"[MeSh] OR delivery[Tiab] OR release[Tiab] OR "drug release"[MeSh] OR "drug liberation"[MeSh] OR "sustained delivery"[Tiab] OR "controlled release*"[Tiab] OR "Delayed-Action Preparations"[Mesh] OR therap*[Tiab] OR therapeutic*[Tiab] OR therapeutics[MeSh] OR treatment[Tiab])
EMBASE	(microneedle*:ti,ab,kw OR 'microneedle array*':ti,ab,kw OR 'microarray patch*':ti,ab,kw OR 'microneedle patch':ti,ab,kw OR 'dissolving microneedle*':ti,ab,kw OR 'dissolving microneedle array*':ti,ab,kw OR 'hydrogel forming microneedle*':ti,ab,kw) NOT ('solid microneedle*':ti,ab,kw OR 'solid microarray*':ti,ab,kw OR 'coat* microneedle*':ti,ab,kw OR 'microneedle coat*':ti,ab,kw OR 'coat* microarray*':ti,ab,kw OR 'hollow microneedle*':ti,ab,kw) AND (nanoparticle*:ti,ab,kw OR nanocapsule*:ti,ab,kw OR nanoemulsion:ti,ab,kw OR nanostructur*:ti,ab,kw OR nanoconjugate*:ti,ab,kw OR nanocrystal*:ti,ab,kw OR 'polymer* nanoparticle*':ti,ab,kw OR 'metal nanoparticle*':ti,ab,kw OR 'polymer* nanocapsule*':ti,ab,kw OR nano*sphere*:ti,ab,kw OR lipo*some*:ti,ab,kw OR nio*some*:ti,ab,kw OR nanolip*:ti,ab,kw OR 'nanolip* particle*':ti,ab,kw OR 'polymer* micelles':ti,ab,kw OR 'solid lipid* nanoparticle*':ti,ab,kw OR nanosystem*:ti,ab,kw OR 'nano*structur* lipid* carrier*':ti,ab,kw OR 'nano*structur* carrier*':ti,ab,kw OR nanocarrier*:ti,ab,kw OR nanovesicle*:ti,ab,kw) AND ('transdermal delivery':ti,ab,kw OR 'transdermal patch*':ti,ab,kw OR 'cutaneous route':ti,ab,kw OR 'cutaneous administration':ti,ab,kw OR 'transcutaneous route':ti,ab,kw OR 'transcutaneous administration':ti,ab,kw OR transcutaneous:ti,ab,kw OR 'transdermal drug delivery':ti,ab,kw OR 'drug delivery':ti,ab,kw OR 'drug delivery system*':ti,ab,kw OR 'delivery system*':ti,ab,kw OR 'drug therapy':ti,ab,kw OR delivery:ti,ab,kw OR release:ti,ab,kw OR 'sustained delivery':ti,ab,kw OR 'controlled release':ti,ab,kw OR therap*:ti,ab,kw OR therapeutic*:ti,ab,kw OR treatment:ti,ab,kw) AND [english]/lim
Web of Science	(TI=((Microneedle* OR microneedle array* OR microarray patch* OR microneedle patch OR dissolving microneedle* OR dissolving microneedle array* OR hydrogel forming microneedle*) NOT (solid microneedle* OR solid microarray* OR coat* microneedle* OR microneedle coat* OR coat* microarray* OR hollow microneedle*)) OR AB=((Microneedle* OR microneedle array* OR microarray patch* OR microneedle patch OR dissolving microneedle* OR dissolving microneedle array* OR hydrogel forming microneedle*) NOT (solid microneedle* OR solid microarray* OR coat* microneedle* OR microneedle coat* OR coat* microarray* OR hollow microneedle*)) OR KP=((Microneedle* OR microneedle array* OR microarray patch* OR microneedle patch OR dissolving microneedle* OR dissolving microneedle array* OR hydrogel forming microneedle*) NOT (solid microneedle* OR solid microarray* OR coat* microneedle* OR microneedle coat* OR coat* microarray* OR hollow microneedle*))) AND (TI=(Nanoparticle* OR nanocapsule* OR nanoemulsion OR nanostructur* OR nanoconjugate* OR nanocrystal* OR polymer* nanoparticle* OR metal nanoparticle* OR polymer* nanocapsule* OR nano*sphere* OR lipo*some* OR nio*some* OR nanolip* OR nanolip* particle* OR polymer* micelles OR solid lipid* nanoparticle* OR nanosystem* OR nano*structur* lipid* carrier* OR nano*structur* carrier* OR nanocarrier* OR nanovesicle*) OR AB=(Nanoparticle* OR nanocapsule* OR nanoemulsion OR nanostructur* OR nanoconjugate* OR nanocrystal* OR polymer* nanoparticle* OR metal nanoparticle* OR polymer* nanocapsule* OR nano*sphere* OR lipo*some* OR nio*some* OR nanolip* OR nanolip* particle* OR polymer* micelles OR solid lipid* nanoparticle* OR nanosystem* OR nano*structur* lipid* carrier* OR nano*structur* carrier* OR nanocarrier* OR nanovesicle*) OR KP=(Nanoparticle* OR nanocapsule* OR nanoemulsion OR nanostructur* OR nanoconjugate* OR nanocrystal* OR polymer* nanoparticle* OR metal nanoparticle* OR polymer* nanocapsule* OR nano*sphere* OR lipo*some* OR nio*some* OR nanolip* OR nanolip* particle* OR polymer* micelles OR solid lipid* nanoparticle* OR nanosystem* OR nano*structur* lipid* carrier* OR nano*structur* carrier* OR nanocarrier* OR nanovesicle*)) AND (TI=(transdermal delivery OR transdermal patch* OR cutaneous route OR cutaneous administration OR transcutaneous route OR transcutaneous administration OR transcutaneous OR transdermal drug delivery OR drug delivery OR drug delivery system* OR delivery system* OR “drug therapy” OR delivery OR release OR sustained delivery OR “controlled release” OR therap* OR therapeutic* OR treatment) OR AB=(transdermal delivery OR transdermal patch* OR cutaneous route OR cutaneous administration OR transcutaneous route OR transcutaneous administration OR transcutaneous OR transdermal drug delivery OR drug delivery OR drug delivery system* OR delivery system* OR “drug therapy” OR delivery OR release OR sustained delivery OR “controlled release” OR therap* OR therapeutic* OR treatment) OR KP=(transdermal delivery OR transdermal patch* OR cutaneous route OR cutaneous administration OR transcutaneous route OR transcutaneous administration OR transcutaneous OR transdermal drug delivery OR drug delivery OR drug delivery system* OR delivery system* OR “drug therapy” OR delivery OR release OR sustained delivery OR “controlled release” OR therap* OR therapeutic* OR treatment))

Search on June 12th, 2021

Table S2 – Description of the assays applied to evaluate external stimuli in the release and therapeutic effect of nanostructured systems associated with dissolving microneedles

External stimulus	Microneedle composition	Nanosystem	Evaluated group/method	Evaluation method	Outcomes	Ref.
Iontophoresis	Chitosan	Nanocomposite (graphene quantum dot - GQD)<br>Composition: Lidocaine hydrochloride or<br>Bovine serum albumin, chitosan, and graphene	Device*: *in chemical*	*In chemical:* release method vs. release method under iontophoresis.<br>Glass and vial, temperature 37 ºC, rotation 100 rpm, medium PBS pH 7.4 or water. Prototyping circuit board: power 9 V 280 mAH nickel–metal hydride battery. Quantification by UV-Vis spectroscopy.	Superior release rate for lidocaine (low molecular weight model drug) and for bovine serum albumin (high molecular weight), compared to the conventional method without application of electric current.	[1]
Magnetic field	PVA	Mesoporous iron oxide nanoraspberry (MION)<br>Composition: mesoporous iron oxide and minoxidil	MION: *in chemical and in vitro*<br><br>Device: *in vivo*	*In chemical:* Field-dependent magnetization curves of MION were evaluated by a superconducting quantum interference device.<br>*In chemical:* release method with and without external magnetic field MION treatment (power 3.2 kW and frequency with 1 MHz).<br><br>*In vivo:* animal model of hair growth experiment - Comparison between MION@MN vs. MION@MN + magnetic field	Superior release amount under magnetic field.<br><br>Faster hair growth with MION@MN under magnetic field treatment.	[2]
NIR	PVA/PVP	Polymeric micelle<br>Composition: Methyl ether poly (ethylene glycol)-poly(β-aminoester), AIE™NIR950 aggregation-induced emission luminogens	Polymeric micelle: *in chemical*<br><br>Device: *in vivo*	*In chemical:* photothermal performance of the polyeric micelles exposed to laser irradiation (808 nm, 1 W cm^2, 5 min). The temperature changes were recorded with an infrared thermal imaging camera.<br><br>*In vivo:* Photothermal effect – Comparison between blank MN and micelle@MN under NIR (808 nm, 1 W cm^2, 5 min). The temperature changes were recorded with an infrared thermal imaging camera.<br>Photothermal therapy in melanoma model - Comparison between micelle@MN under NIR 0.5 and 24 h post-administration.	Temperature increase under NIR.<br><br>Significant temperature increased only for micelle@MN compared to blank MN. Tumor reduction favored under laser irradiation.	[3]
NIR	Hyaluronic acid,<br>HP-β-CD,<br>PVP	Nanoparticle<br>Composition: PLGA, DOTAP, α-tocopheryl succinate, indocyanine green, and paclitaxel	Device: *in chemical* and *in vivo*	*In chemical*: Photothermal property – comparison between blank MN and nanoparticles@MN under NIR (808 nm, 0.35 W cm^2, 5 min, 3 cycles on/off). The temperature changes were recorded with an infrared thermal imaging camera.<br><br>*In vivo:* Photothermal effect – Comparison between blank MN and nanoparticles@MN under NIR (808 nm, 0.35 W cm^2, 5 min). The temperature changes were recorded with an infrared thermal imaging camera.<br>Photothermal therapy in melanoma model - Comparison between blank MN nanoparticles@MN under NIR.	Significant temperature increased only for nanoparticles@MN compared to blank MN.<br><br>Significant temperature increased only for nanoparticles@MN compared to blank MN. Tumor reduction favored under laser irradiation for paclitaxel-indocyanine green nanoparticle@MN.	[4]

NIR	Hyaluronic acid/PVP	Solid lipid nanoparticle<br>Composition: Pluronic F-68, cetyl Palmitate, tricaprin, IR-780 (photothermal agent), and paclitaxel	Solid lipid nanoparticle: *in chemical and in vitro*<br><br>Device: *in vivo*	*In chemical*: photothermal performance of the solid lipid nanoparticles and IR-780 solution exposed to laser irradiation (808 nm, 1 W cm^2, 5 min), measured by fluorescence.<br>*In chemical*: Release method vs. release method under NIR. Centrifuge tubes, temperature 50 ºC, medium PBS + 1% polyoxyethylated castor oil, laser irradiation (808 nm, 1 W cm^2, 5 min). Quantification by HPLC.<br><br>*In vitro:* Laser-triggered intracellular drug release, melanoma cell line under laser irradiation (808 nm, 0.5 or 1 W cm^2, 5 min). Fluorescence captured by confocal laser scanning microscopy.<br><br>*In vivo*: Photothermal effect – Comparison between blank MN and solid lipid nanoparticle@MN under NIR (808 nm, 1 W cm^2, 5 min). The temperature changes were recorded with an infrared thermal imaging camera.<br>Photothermal therapy in melanoma model - Comparison between blank MN nanoparticles@MN under NIR (plus control groups).	Increase in temperature and the amount of released drug when exposed to laser irradiation.<br><br>Increased cytotoxicity under laser irradiation.<br><br>Significant temperature increased only for solid lipid nanoparticle@MN compared to blank MN. Tumor reduction favored under laser irradiation for solid lipid nanoparticle@MN.	[5]
NIR	Hyaluronic acid	Nanoparticle<br>Composition: 5-luorouracil, indocyanine green, and monomethoxy-poly (ethylene glycol)-polycaprolactone	Nanoparticle: *in chemical and in vitro*<br><br>Device: *in chemical* and *in vivo*	*In chemical:* release method vs. release method under laser irradiation. Dialysis bags, temperature 37 ºC, rotation 100 rpm, medium PBS pH 7.4, laser irradiation (808 nm, 1.5 W cm^2, 5 min). Quantification by HPLC.<br><br>*In vitro*: Photothermal effect in human epidermoid cancer and melanoma cell lines. Laser irradiation: 808 nm, 0.5 or 1.5 W cm^2, 5 min. The effect was observed under fluorescence microscopy.<br><br>Device – *in chemical:* photothermal performance of MN was measured under NIR (at same parameters), comparison between blank MN and nanoparticles@MN. The temperature changes were recorded with an infrared thermal imaging camera.<br><br>*In vivo*: Photothermal effect – Comparison between blank MN and nanoparticles@MN under NIR (808 nm, 1.5 W cm^2, 5 min). The temperature changes were recorded with an infrared thermal imaging camera.<br>Photothermal therapy in epidermoid cancer and melanoma models - Comparison between blank MN nanoparticles@MN under NIR (plus control groups).	Increase in temperature and the amount of released drug when exposed to laser irradiation.<br><br>Increased cytotoxicity under laser irradiation.<br><br>Significant temperature increased only nanoparticles@MN compared to blank MN. Tumor reduction favored under laser irradiation for nanoparticles@MN.	[6]
NIR	Hyaluronic acid/PVP	Nanoparticle<br>Composition: MIL-88 (ferric ions and 2-aminoterephthalic	Nanoparticle: *in chemical* and *in vitro*	*In chemical*: photothermal performance of the nanoparticles (at different concentrations) and indocyanine green solution	Increase in temperature and the amount of released drug when exposed to laser	[7]

		acid (BDC-NH_2), ZIF-8 (composed of zinc ions and 2-methyl imidazole (2-MeIM), indocyanine green, and doxorubicin	Device: *in vivo*	exposed to laser irradiation (808 nm, 5 min). The temperature changes were recorded with an infrared thermal imaging camera.<br>*In chemical*: Release method vs. release method under NIR. Assay parameters: Temperature 50 ºC, medium PBS (pH 5.0, 6.0, and 7.0), laser irradiation (808 nm, 5 min). Quantification by fluorescence spectroscopy.<br><br>*In vitro*: Photothermal effect in breast cancer cell lines. Laser irradiation: 808 nm, 1 W cm^2, 5 min. The effect was observed by the cytotoxicity value.<br><br>*In vivo*: Photothermal therapy in breast cancer model - Comparison between nanoparticles@MN and I.V. nanoparticles under NIR (plus control groups).	irradiation. Release enhancement at acidic pH.<br><br>Increased cytotoxicity under laser irradiation.<br><br>Significant temperature increased after nanoparticles administration by transcutaneous and I.V. administration. The tumor reduction favored under laser irradiation for nanoparticles@MN, when compared to the intravenous route.	
Photodynamic therapy	Methacrylated hyaluronic acid	Nanoparticle<br>Composition: anti-CTLA4 antibody (aCTLA4), photosensitizer zinc phthalocyanine, acetal modified dextran, PVA	Device: *in vivo*	*In vivo*: Photodynamic therapy in breast cancer model - Comparison between nanoparticles@MN and antibody-nanoparticles@MN under laser (plus control groups), 600 nm for 10 min.	Significant reduction of tumor volume for the group treated with antibody-nanoparticles@MN.	[8]

NIR: Near-infrared-light; PVA: poly (vinyl alcohol); PVA/PVP: poly (vinyl alcohol)/polyvinylpyrrolidone; HP-β-CD: hydroxypropyl-β-cyclodextrin; PVP: polyvinylpyrrolidone; PLGA: poly (D, L-lactide-co-glycolide); DOTAP: 1,2-dioleoyl-3-trimethylammonium-propane; MION: Mesoporous iron oxide nanoraspberry; MN: Microneedle; I.V.: intravenous route. *Device: polymeric microneedle containing the nanostructured system.

FUGURES

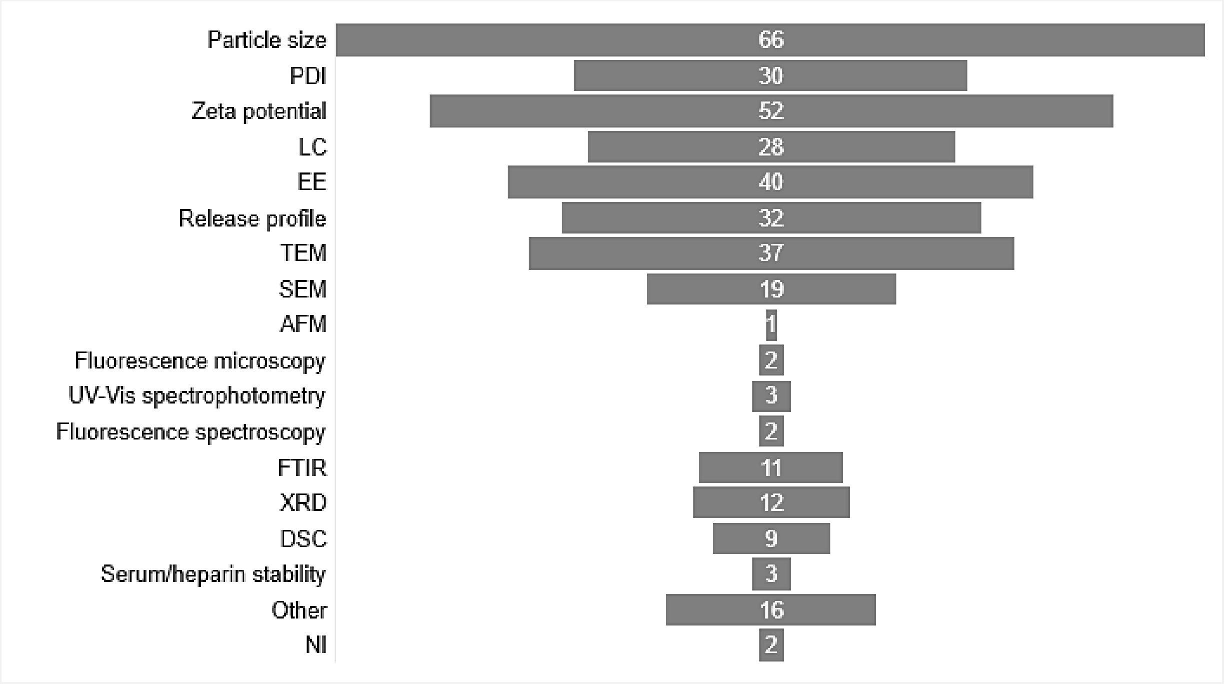

Figure S1 - Characterization tests of nanostructured systems prior to inclusion into the polymeric matrix of the microneedle by number of studies included in the review. PDI: polydispersity index; LC: loading capacity; EE: encapsulation/entrapment efficiency; TEM: transmission electron microscopy; SEM: scanning eletronic microsocpy; AFM: atomic force microscopy; FTIR: fourier transform infrared spectroscopy; XRD: X-ray powder diffraction; DSC: differential scanning calorimetry; NI: not informed.

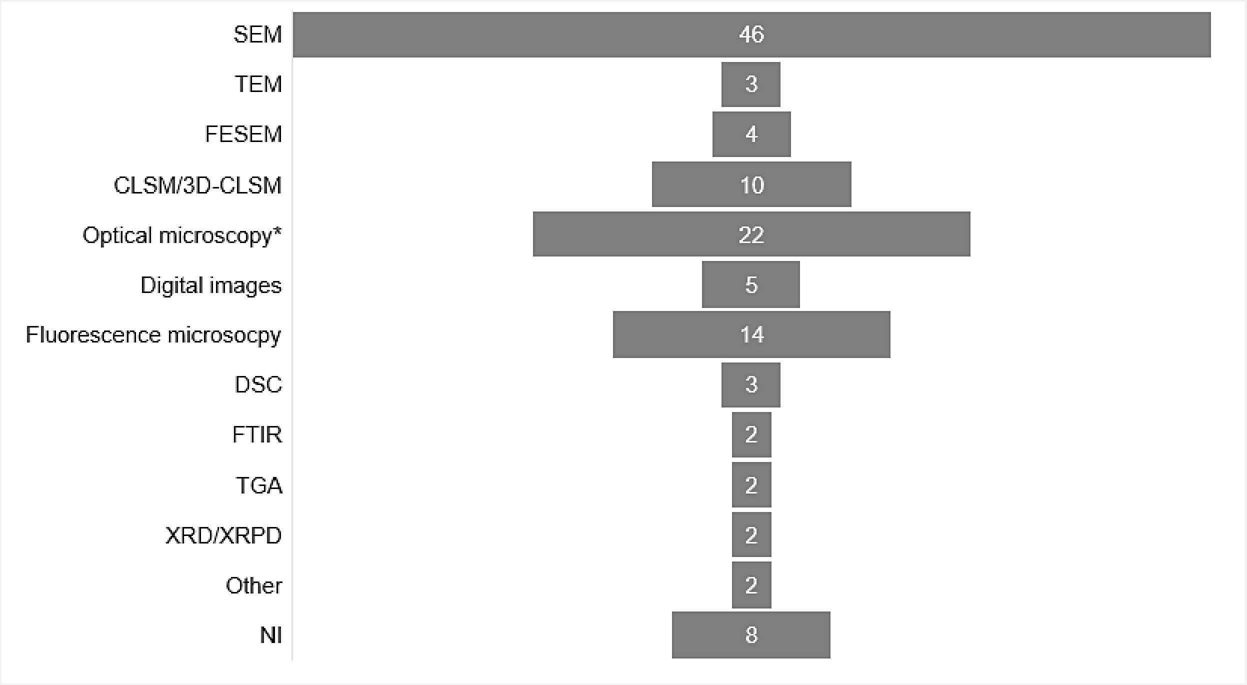

Figure S2 - Characterization tests of MN device by number of studies included in the review. SEM: scanning eletronic microsocpy; FESEM: field emission scanning eletronic microsocpy; TEM: transmission electron microscopy; CLSM/3D-CLSM: Confocal laser scanning microscopy; DSC: differential scanning calorimetry; FTIR: fourier transform infrared spectroscopy; TGA: Thermal gravimetric analysis; XRD/ XRPD: X-ray diffraction/X-ray powder diffraction; NI: not informed. *Optical microscopy: corresponding to bright field microscopy, optical microscopy, and digital microscopy.

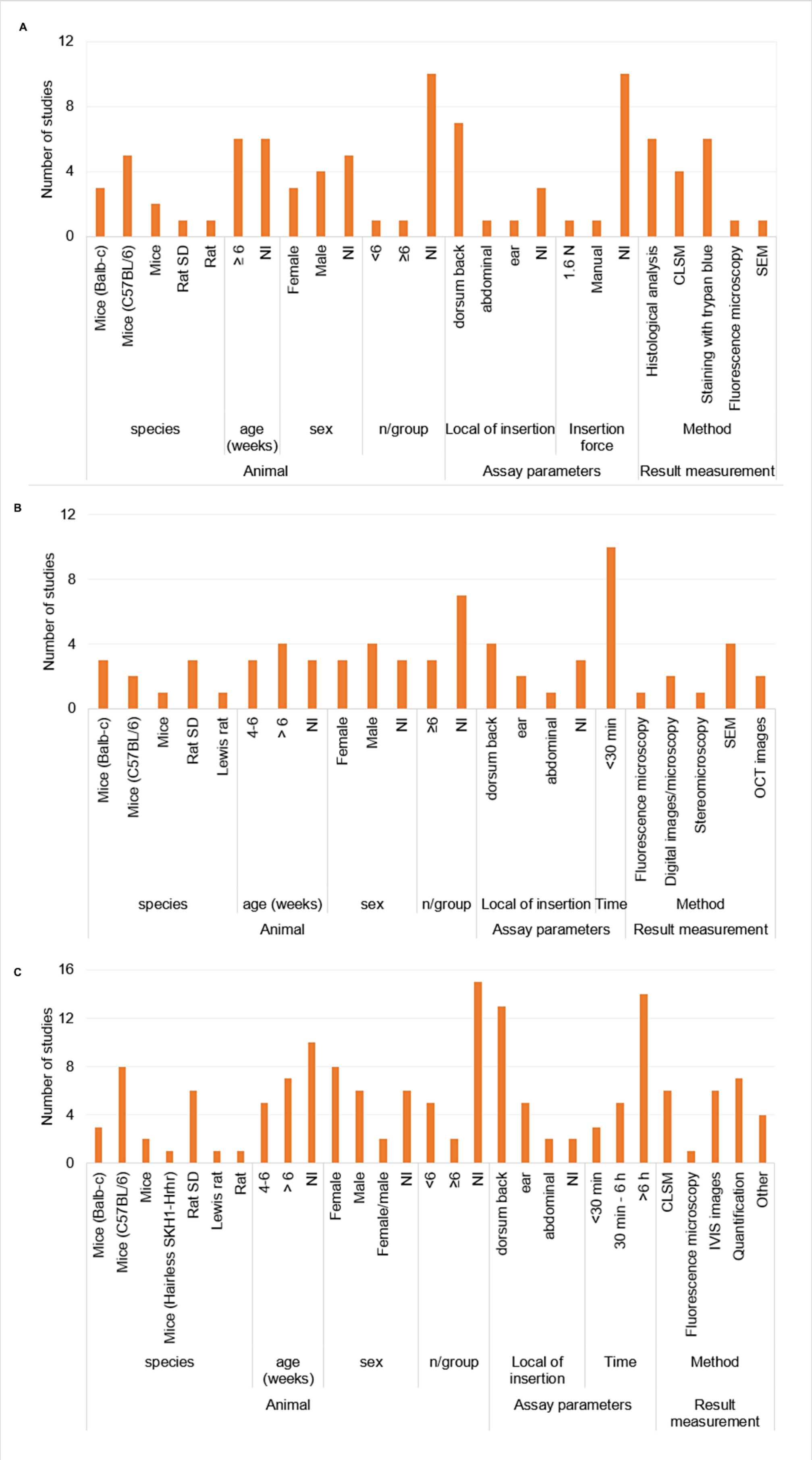

Figure S3 - General parameters of *in vivo* skin insertion assay (A), *in vivo* skin dissolution assay (B), and in vivo skin release/permeation/distribution assay (C) by number of studies. Rat SD: Sprague Dawley; NI: not informed; IVIS*: In vivo* imaging system; CLSM: confocal laser scanning microscopy; SEM: scanning eletronic microsocpy; OCT: optical coherence tomography.

Supplementary material references

1. Justin, R.; Román, S.; Chen, D.; Tao, K.; Geng, X.; Grant, R.T.; MacNeil, S.; Sun, K.; Chen, B. Biodegradable and conductive chitosan-graphene quantum dot nanocomposite microneedles for delivery of both small and large molecular weight therapeutics. *RSC Adv.* **2015**, *5*, 51934–51946, doi:10.1039/c5ra04340a.
2. Fang, J.H.; Liu, C.H.; Hsu, R.S.; Chen, Y.Y.; Chiang, W.H.; Wang, H.M.D.; Hu, S.H. Transdermal composite microneedle composed of mesoporous iron oxide nanoraspberry and PVA for androgenetic alopecia treatment. *Polymers (Basel).* **2020**, *12*, 1–14, doi:10.3390/polym12061392.
3. Wei, S.; Quan, G.; Lu, C.; Pan, X.; Wu, C. Dissolving microneedles integrated with pH-responsive micelles containing AIEgen with ultra-photostability for enhancing melanoma photothermal therapy. *Biomater. Sci.* **2020**, *8*, 5739–5750, doi:10.1039/d0bm00914h.
4. Peng, T.; Huang, Y.; Feng, X.; Zhu, C.; Ma, X.; Wang, X.; Bai, X.; Pan, X.; Wu, C. Dissolving microneedles loading TPGS biphasic functionalized PLGA nanoparticles for efficient chemo-photothermal combined therapy of melanoma. *Adv. Ther.* **2020**, *3*, 1–11, doi:10.1002/adtp.201900190.
5. Qin, W.; Quan, G.; Sun, Y.; Chen, M.; Yang, P.; Feng, D.; Wen, T.; Hu, X.; Pan, X.; Wu, C. Dissolving microneedles with spatiotemporally controlled pulsatile release nanosystem for synergistic chemo-photothermal therapy of Melanoma. *Theranostics* **2020**, *10*, 8179–8196, doi:10.7150/thno.44194.
6. Hao, Y.; Chen, Y.W.; He, X.L.; Yang, F.; Han, R.X.; Yang, C.L.; Li, W.; Qian, Z.Y. Near-infrared responsive 5-fluorouracil and indocyanine green loaded MPEG-PCL nanoparticle integrated with dissolvable microneedle for skin cancer therapy. *Bioact. Mater.* **2020**, *5*, 542–552, doi:10.1016/j.bioactmat.2020.04.002.
7. Wu, B.; Fu, J.; Zhou, Y.; Luo, S.; Zhao, Y.; Quan, G.; Pan, X.; Wu, C. Tailored core–shell dual metal–organic frameworks as a versatile nanomotor for effective synergistic antitumor therapy. *Acta Pharm. Sin. B* **2020**, *10*, 2198–2211, doi:10.1016/j.apsb.2020.07.025.
8. Chen, S.X.; Ma, M.; Xue, F.; Shen, S.; Chen, Q.; Kuang, Y.; Liang, K.; Wang, X.; Chen, H. Construction of microneedle-assisted co-delivery platform and its combining photodynamic/immunotherapy. *J. Control. Release* **2020**, *324*, 218–227, doi:10.1016/j.jconrel.2020.05.006.
